# Combining shrinkage and sparsity in conjugate vector autoregressive models

**DOI:** 10.1002/jae.2807

**Published:** 2021-01-13

**Authors:** Niko Hauzenberger, Florian Huber, Luca Onorante

**Affiliations:** ^1^ Department of Economics, Salzburg Centre of European Union Studies University of Salzburg Salzburg Austria; ^2^ Joint Research Centre European Commission Ispra Italy

## Abstract

Conjugate priors allow for fast inference in large dimensional vector autoregressive (VAR) models. But at the same time, they introduce the restriction that each equation features the same set of explanatory variables. This paper proposes a straightforward means of postprocessing posterior estimates of a conjugate Bayesian VAR to effectively perform equation‐specific covariate selection. Compared with existing techniques using shrinkage alone, our approach combines shrinkage and sparsity in both the VAR coefficients and the error variance–covariance matrices, greatly reducing estimation uncertainty in large dimensions while maintaining computational tractability. We illustrate our approach by means of two applications. The first application uses synthetic data to investigate the properties of the model across different data‐generating processes, and the second application analyzes the predictive gains from sparsification in a forecasting exercise for U.S. data.

## 1 INTRODUCTION

This paper deals with estimating vector autoregressive (VAR) models of the following form:

(1) $$y_{t}=A_{1}y_{t-1}+\ldots +A_{p}y_{t-p}+C+\varepsilon_{t},$$

where $y_{t}={\left(y_{1t},\ldots ,y_{mt}\right)}^{\prime }$ denotes an *m*‐dimensional vector of time series measured in time t=1,…,T, $A_{j}\;(j=1,\ldots ,p)$ is an (*m* × *m*)‐dimensional matrix of coefficients associated with the *j*th lag of **y**_*t*_, **C** is an *m*‐dimensional intercept vector, and $\varepsilon_{t}∼N(0,\Sigma )$ is a Gaussian shock vector with zero mean and an (*m* × *m*)‐dimensional variance‐covariance matrix **Σ**. For further convenience, let $a=\text{vec}\{{\left(A_{1},\ldots ,A_{p},C\right)}^{\prime }\}$ denote a vector of dimension *k* = *m*(*mp* + 1) of vectorized coefficients with $a_{i}\;(i=1,\ldots ,k)$ denoting its *i*th element. This model class has been extensively used for forecasting and policy analysis in central banks (see Alessi, Ghysels, Onorante, et al., 2014) as well as a natural starting point for unveiling stylized time series facts in order to estimate theoretical models (see, inter alia, Hall, Inoue, Nason, & Rossi, 2012).

Conditional on the first *p* observations, estimation of the model in Equation (1) can be carried out using ordinary least squares (OLS). In this case, however, overfitting issues arise, translating into imprecise out‐of‐sample forecasts. As a potential solution, the Bayesian literature uses informative priors to push the system toward a prior model. For instance, the widely used Minnesota prior assumes that the elements in **y**_*t*_ follow a random walk a priori (Doan, Litterman, & Sims, 1984; Litterman, 1986; Giannone, Lenza, & Primiceri, 2015). Theoretically inspired restrictions stemming from structural models can also be used to inform parameter estimates and thus improve inference (Ingram & Whiteman, 1994; Del Negro & Schorfheide, 2004). The key feature of these priors is that they are conjugate, implying that the likelihood and the prior feature the same distributional form. This yields closed‐form solutions for key posterior quantities and, if simulation‐based techniques are necessary, greatly improves estimation speed.

In VARs, conjugacy requires that each equation in the system features the same set of predictors, potentially leading to model misspecification (see, e.g., George, Sun, & Ni, 2008; Koop, 2013). This translates into a Kronecker structure in the likelihood, prior, and the resulting posterior distribution, implying that inversion of the posterior variance–covariance matrix of the coefficients is computationally cheap. By contrast, models based on nonconjugate priors allow for different predictors across equations by specifying the prior on the VAR coefficients independently of **Σ**. This, however, is computationally much more demanding, because the convenient Kronecker structure is lost. 1

Apart from reduced flexibility in terms of covariate selection across equations, typical shrinkage priors push many VAR coefficients toward zero. Under continuous shrinkage priors, however, this implies that the probability of observing a coefficient that exactly equals zero is zero (see, e.g., Bhattacharya, Pati, Pillai, & Dunson, 2015; Carvalho, Polson, & Scott, 2010; Griffin & Brown, 2010; Huber & Feldkircher, 2019; Huber, Koop, & Onorante, 2020). Spike and slab priors allow for shrinking coefficients exactly to zero. These priors rely on an additional set of auxiliary binary indicators that determine whether a coefficient is zero or nonzero. In large models with *k* covariates (such as the VAR models we consider), estimating these indicators is cumbersome because the number of potential models is 2^*k*^. In such a situation, Markov chain Monte Carlo (MCMC) techniques often fail to explore this vast model space and convergence issues arise (see Polson & Scott, 2010).

In recent contributions, Hahn and Carvalho (2015) and Ray and Bhattacharya (2018) propose a way to circumvent insufficient shrinkage/variable selection in light of an increasing amount of predictors (i.e., the curse of dimensionality problem). They estimate a large‐scale regression model under a suitable shrinkage prior and then post‐process a point estimator (the posterior mean) such that the distance between the fit of the model based on the shrinkage prior and a model based on a sparse estimator (i.e., with coefficients set equal to zero) is minimized while accounting for a penalty term that depends on the *L*1‐norm of the coefficients. This approach, labeled decoupled shrinkage and selection (DSS), yields a sparse estimator and is analogous to solving a lasso‐type problem. One key disadvantage, however, is the nonautomatic nature of this approach. A semiautomatic approach that is similar in nature is described in Ray and Bhattacharya (2018). In this framework, an optimization problem to efficiently set coefficients associated with irrelevant predictors to zero is solved. But instead of performing cross validation, this task depends only on a single tuning parameter that needs to be chosen by the researcher. These techniques that combine shrinkage and sparsity have been shown to work well in a wide range of applications ranging from finance (Puelz, Hahn, & Carvalho, 2017; 2020) to macroeconomics (Huber et al., 2020).

In this paper, we deal with both issues discussed above by proposing a fully conjugate VAR model coupled with the prior proposed in Kadiyala and Karlsson (1997) and Koop (2013). Our model allows for different covariates across equations, sparsity in terms of the VAR coefficients, and data‐based zero restrictions on the covariance parameters in **Σ**. Instead of post‐processing posterior mean/median estimates, we follow Huber et al. (2020) in sparsifying each draw from the posterior distribution. This yields an approximate posterior distribution for a sparse vector of coefficients that can be used for uncertainty quantification. The key advantage is that this significantly reduces estimation uncertainty if the data‐generating process is sparse. For example, if there is strong evidence that *a*_*i*_ equals zero, our proposed framework is capable of selecting this restriction consistently across different draws from the posterior distribution of *a*_*i*_. This implies that the posterior variance of *a*_*i*_, in the limiting case that each draw of *a*_*i*_ is set to zero, also equals zero. In terms of forecasting, the reduced estimation uncertainty could then lead to more precise predictions, especially in situations where *k* is large.

The merits of our proposed approach are illustrated by means of two applications. In the first application, we use synthetic data obtained from a set of different data generating processes (DGPs) that differ in terms of sparsity, model size, and number of observations. Across DGPs, we find that (i) our framework successfully detects zero values in both the VAR coefficients and the error variance–covariance matrices and (ii) it outperforms other Bayesian VARs (BVARs) in terms of root mean square errors (RMSEs). In the second application, we forecast U.S. output, inflation, and short‐term interest rates using the dataset compiled in McCracken and Ng (2016). We find that applying the additional sparsification step often improves point and density forecasts. In turbulent times (such as the period of the global financial crisis), however, our results also show that using sparsification could harm the accuracy of density forecasts by underestimating the predictive variance. Nevertheless, these accuracy losses are never substantial and forecasts are still competitive.

The remainder of the paper is structured as follows. Section 2 introduces the conjugate Bayesian VAR, whereas Section 3 discusses the techniques to achieve sparsity in this model. Section 4 documents model features when applied to synthetic data. Section 5 summarizes the results of the forecast exercise with real data. Section 6 concludes the findings of the paper, and the Supporting Information provides details on data and additional empirical results.

## 2 CONJUGATE BAYESIAN INFERENCE IN VAR MODELS

Before discussing prior implementation, it is worth noting that Equation (1) can be rewritten as a standard regression model,

(2) $$y_{t}=(I_{m}⊗x_{t}^{\prime })a+\varepsilon_{t},$$

with $x_{t}={\left(y_{t-1}^{\prime },\ldots ,y_{t-p}^{\prime },1\right)}^{\prime }$ denoting an *n*(= *pm* + 1)‐dimensional vector of explanatory variables. In terms of full‐data matrices **Y** (with *t*th row $y_{t}^{\prime }$) and **X** (with *t*th row $x_{t}^{\prime }$), the model reads

(3) Y=XA+E,

where $A={\left(A_{1},\ldots ,A_{p},C\right)}^{\prime }$ and **E** is a (*T* × *m*)‐dimensional matrix of stacked shocks with *t*th row given by $\varepsilon_{t}^{\prime }$.

The model in Equation (3) features *k* parameters in ***a*** and *w* = *m*(*m* + 1)/2 free parameters in **Σ**. If *m* and *p* become large, the number of parameters sharply increases, making precise estimation almost impossible. To deal with this issue, Bayesian econometricians rely on informative priors. This implies that more weight is placed on the prior and the resulting posterior distribution of ***a*** and **Σ** will be strongly influenced by the prior model (such as the random walk).

The general form of the conjugate prior in VARs assumes dependence between ***a*** and **Σ** and is given by

(4) $$a|\Sigma ∼N\left(a_{0},\Sigma ⊗V_{0}(\delta )\right).$$

***a***_0_ denotes a *k*‐dimensional prior mean vector and **V**_0_(**δ**) is a prior variance–covariance matrix that depends on a lower dimensional set of *q* hyperparameters in **δ**. In what follows, we assume that **y**_*t*_ is stationary, and thus, a common choice for the prior mean would be $a_{0}=0$.

For **V**_0_(**δ**), we use a variant of the conjugate Minnesota prior (Kadiyala & Karlsson, 1997; Koop, 2013) that can be implemented using a set of dummy observations (Bańbura, Giannone, & Reichlin, 2010) that are then concatenated to **Y** and **X**:

$$\underline{Y}=\left(\begin{array}{c} \text{diag}(\phi_{1}{\hat{\sigma }}_{1},\ldots ,\phi_{m}{\hat{\sigma }}_{m})/\theta_{1}\\ 0_{m(p-1)\times m}\\ diag({\hat{\sigma }}_{1},\ldots ,{\hat{\sigma }}_{m})\\ 0_{1\times m} \end{array}\right),\;\underline{X}=\left(\begin{array}{cc} J_{p}⊗\text{diag}({\hat{\sigma }}_{1},\ldots ,{\hat{\sigma }}_{m})/\theta_{1} & \;0_{mp\times 1}\\ 0_{m\times mp} & \;0_{m\times 1}\\ 0_{1\times mp} & \;\pi^{-1/2} \end{array}\right),$$

with $V_{0}(\delta )={\left({\underline{X}}^{\prime }\underline{X}\right)}^{-1}$. Here, $J_{p}={\left(1,\ldots ,p\right)}^{\prime }$, *π* is a hyperparameter that determines the prior variance on the intercepts, and $\phi_{i}\;(i=1,\ldots ,m)$ represents the prior mean associated with the coefficient on the first own lag of a given variable (which is consequently set equal to zero). In addition, we let ${\hat{\sigma }}_{i}^{2}$ denote the OLS variances obtained by estimating *m* univariate AR(*p*) models for each element in **y**_*t*_. Finally, *θ*_1_ is a hyperparameter that controls the overall tightness of the prior. Lower values of *θ*_1_ imply a stronger prior belief, effectively pushing the elements in ***a*** toward ***a***_0_. For *π*, we simply set it equal to a large value to render the prior on the intercept weakly informative. This prior setup implies that $\delta ={\left(\theta_{1},\pi \right)}^{\prime }$ is a two‐dimensional vector.

The final ingredient is a conjugate prior on **Σ**. Here, conjugacy implies a prior on **Σ** that does not depend on ***a*** and follows an inverted Wishart distribution:

(5) $$\Sigma ∼W^{-1}(s_{0},S_{0}).$$

We let *s*_0_ denote the prior degrees of freedom and **S**_0_ a prior scaling matrix. The main shortcoming of this prior choice is that shrinking *specific* covariances in **Σ** to zero is impossible. For instance, even if there exists significant evidence that contemporaneous relations across elements in **y**_*t*_ equal zero, this prior is not capable of selecting such restrictions and the resulting posterior estimate of **Σ** (and of its inverse) will be nonsparse.

In the general case (i.e., with any form of the prior hyperparameters), one can show that the conditional posterior of ***a*** is given by

(6) $$a|\Sigma ,Y,X∼N(\text{vec}(\bar{A}),\Sigma ⊗\bar{V}),$$

with

(7) $$\bar{V}={\left(X^{\prime }X+V_{0}{\left(\delta \right)}^{-1}\right)}^{-1},$$

(8) $$\bar{A}=\bar{V}\left(X^{\prime }Y+V_{0}{\left(\delta \right)}^{-1}A_{0}\right).$$

Here, we let **A**_0_ denote an (*n* × *m*)‐dimensional matrix reshaped such that $a_{0}=\text{vec}(A_{0})$.

Using the Minnesota dummies, the posterior moments can be obtained by applying Theil and Goldberger (1961) mixed estimation:

$$\bar{V}={\left({\bar{X}}^{\prime }\bar{X}\right)}^{-1},\;\bar{A}=\bar{V}\;{\bar{X}}^{\prime }\bar{Y},$$

where $\bar{Y}={\left(Y,\underline{Y}\right)}^{\prime }$ and $\bar{X}={\left(X,\underline{X}\right)}^{\prime }$ denote full‐data matrices augmented with dummy observations.

Under the prior in Equation (5), the posterior distribution also follows an inverted Wishart distribution,

(9) $$\Sigma |Y,X∼W^{-1}(s_{1},S_{1}).$$

The posterior degrees of freedom are denoted by $s_{1}=T+s_{0}$, and $S_{1}={\left(\bar{Y}-\bar{X}\;\bar{A}\right)}^{\prime }(\bar{Y}-\bar{X}\;\bar{A})$ represents the (*m* × *m*)‐dimensional posterior scaling matrix.

A key advantage of conjugacy is the Kronecker structure in Equation (6), which implies that $V=\Sigma ⊗\bar{V}$ is a block‐diagonal matrix and computing the inverse or the Cholesky factor is computationally cheap. By contrast, if V were a full (*k* × *k*) matrix, computation would quickly become cumbersome and impossible even for moderate values of *m* and *p*. One further advantage of the conjugate prior is that the one‐step‐ahead predictive density and the marginal likelihood (ML) are available in closed form (see, for instance, Zellner, 1985). This implies that if interest centers on one‐step‐ahead forecasts, no posterior simulation is required. 2

Unfortunately, the conjugate prior has two important shortcomings. First, each equation must include the same set of covariates (see Equation (2)), a feature that could be unappealing if the researcher wishes to introduce theoretically motivated restrictions across equations. Second, the structure of the prior variance–covariance matrix implies that for each equation j=1,…,m, the prior variance is given by $\sigma_{jj}^{2}V_{0}(\delta )$, with $\sigma_{jj}^{2}$ denoting the (*j*, *j*)th element of **Σ**. One consequence of this is that across equations, the prior variances are proportional to each other. This implies that it is not possible to discriminate between coefficients on own (which we define as lags of the *j*th endogenous variable in equation *j*) and other (defined as the lags of the *i*th endogenous variable for *i* ≠ *j* within equation *j*) lags. The methods we discuss in the next section allow for different treatment of own and other lags in a simple way.

## 3 ACHIEVING SPARSITY IN VAR MODELS

### 3.1 Overview of the problem

From a forecaster's perspective, heavily parameterized models, such as large‐scale VARs, have another important shortcoming. The continuous shrinkage prior described in Section 2 implies that the probability of observing exact zeros in ***a*** equals zero. One could ask whether it makes a big difference to zero out different *a*_*i*_'s as opposed to setting them close to zero. Setting them close but not exactly to zero essentially implies that there exists a lower bound of accuracy one can achieve under the specific prior distribution (Huber et al., 2020). For small‐scale systems, this has negligible implications on predictive accuracy. However, if *k* is large (i.e., of order 1000 or 10,000), parameter uncertainty adds up and potentially dominates the predictive variance. To see this point, notice that under the conjugate prior, the one‐step‐ahead predictive density follows a multivariate *t*‐distribution (see Koop, 2013) with predictive variance given by

(10) $$\mathrm{Var}(y_{T+1}|Y,X)=\frac{1}{s_{1}-2}\left(1+\overset{n}{\underset{i=1}{\sum }}\overset{n}{\underset{j=1}{\sum }}\left(x_{iT+1}x_{jT+1}v_{ij}\right)\right)S_{1},$$

with Var(•) denoting the variance operator, *x*_*iT* + 1_ the *i*th element of **x**_*T* + 1_, and *v*_*ij*_ referring to the (*i*, *j*)th element in $\bar{V}$.

Here, it can be seen that the predictive variance depends on the variance of the reduced‐form shocks in **ε**_*T* + 1_ and parameter uncertainty arising from the term in the parenthesis. Equation (10) indicates that if *n* increases, posterior uncertainty rises even if the *v*_*ij*_'s associated with irrelevant predictors are small. This point clearly highlights the difference between sparsity and shrinkage, namely, the fact that under a sparse model, *v*_*ij*_ would be equal to zero if and only if the relevant predictor is excluded from the model. In the next subsections, we will show how this lower bound on accuracy (determined by small but nonzero values of *v*_*ij*_) can be removed.

Equation (10), moreover, highlights that the uncertainty associated with coefficients potentially adds up in large‐scale models and the variance implied by the reduced‐form shocks further influences the predictive variance. Without additional restrictions, these two sources can act in opposite directions. In the case of a large‐scale model, the variance–covariance matrix might be underestimated due to overfitting while uncertainty surrounding parameter estimates is too large. The second effect is mainly driven by the fact that in VAR models and with standard macroeconomic datasets, covariates are often highly correlated, and this, in combination with insufficient shrinkage, inflates variance estimates of the regression coefficients. Since these two sources play a crucial role in forming accurate forecasts, it is imperative to treat both of them carefully.

### 3.2 Achieving sparsity on the VAR coefficients

Since obtaining a sparse representation of ***a*** is unfeasible in high dimensions due to the necessity to explore a model space of cardinality 2^*k*^, we follow a different route that combines shrinkage and sparsity. Our approach follows Hahn and Carvalho (2015) and Ray and Bhattacharya (2018) and is based on manipulating an estimator $\hat{a}$ ex‐post by solving the following optimization problem:

(11) $${\hat{a}}^{*}=\underset{\alpha }{\text{arg min}}\left\{\frac{1}{2}{||\left(Z\hat{a}-Z\alpha \right)||}_{2}^{2}+\overset{k}{\underset{j=1}{\sum }}\kappa_{j}|\alpha_{j}|\right\},$$

with $Z=(I_{m}⊗X)$, **α** being a sparse *k*‐dimensional vector and ${||m||}_{2}$ denoting the Euclidean norm of a vector **m**. Equation (11) consists of two components. The first part measures the Euclidean distance between the fit of an unrestricted model, estimated using the shrinkage prior described in Section 2, and a sparse model determined by **α**. The second part is a penalty term that penalizes nonzero values in **α**, with *κ*_*j*_ denoting variable‐specific penalties. In light of large *k* (which is almost always the case in moderately‐sized VARs), choosing the tuning parameters *κ*_*j*_ by means of cross‐validation becomes computational prohibitive.

To circumvent the necessity to employ cross‐validation, we adopt the signal adaptive variable selection (SAVS) estimator proposed in Ray and Bhattacharya (2018). We rewrite Equation (11) in terms of the *j*th column of **Z**, **Z**_*j*_, and solve the optimization problem in Equation (11) for each covariate individually, adopting the coordinate descent algorithm (Friedman, Hastie, Höfling, & Tibshirani, 2007). This yields the following solution to the optimization problem in Equation (11), 3

(12) $${\hat{a}}_{j}^{*}=\text{sign}({\hat{a}}_{j})\;||Z_{j}|{|}^{-2}{\left(|{\hat{a}}_{j}|\;||Z_{j}|{|}^{2}-\kappa_{j}\right)}_{+},$$

for j=1,…,k, with sign(*c*) returning the sign of a real number *c* and ${\left(c\right)}_{+}=\max\{c,0\}$ returns *c* if it is positive or zero if *c* ≤ 0.

The penalty term is set as follows:

(13) $$\kappa_{j}=\frac{\lambda }{|{\hat{a}}_{j}{|}^{\zeta }}.$$

*κ*_*j*_ depends on the nonsparse estimate ${\hat{a}}_{j}$ and two hyperparameters *λ* > 0 and *ζ* ≥ 1. Setting *ζ* ≥ 1 implies that smaller values of ${\hat{a}}_{j}$ receive a larger penalty and are likely to be zeroed out by the SAVS algorithm.

A typical choice, proposed in Ray and Bhattacharya (2018), sets λ=1 and ζ=2. We show below that, in simulations, this choice works well. The approach stipulated in Hahn and Carvalho (2015) is obtained by setting ζ=1 while inferring *λ* by visually inspecting the posterior output. More specifically, Hahn and Carvalho (2015) suggested choosing *λ* such that the variation‐explained by a sparsified linear predictor (which is akin to a standard *R*^2^) statistically equals that of the nonsparsified model. For carrying out structural analysis, this poses no problem because it needs to be done only once. However, if the researcher is interested in assessing forecasting accuracy, this procedure has to be repeated sequentially over a hold‐out period. This makes the nonautomatic nature of the approach problematic.

As stated above, the natural conjugate Minnesota prior is not capable of discriminating between the lags of own and other variables. In this paper, we modify the SAVS estimator accordingly. In what follows, we replace *λ* with a lag‐wise parameter that increases the weight associated with coefficients on higher‐order lags of **y**_*t*_ and impose a stronger penalty on coefficients related to the other lags within a given equation. Moreover, we do not sparsify the diagonal elements of **A**_1_. These parameters are specified for each **A**_*l*_ such that

(14) $$\lambda_{l,ij}=\left\{\begin{array}{ll} \lambda \;{\left(l-1\right)}^{2} & \text{if},\;i=j\\ \lambda \;l^{2} & \text{if}\;i\neq j, \end{array}\right.$$

for l=1,…,p;i=1,…,m;j=1,…,m. Here, we assume that *λ* is some lag‐invariant scaling parameter and *λ*_*l*, *ij*_ increases quadratically with the lag order. Note that for the first, own lag of a given equation, we set the penalty equal to zero. This captures the notion that this covariate is crucial and its coefficient should never be set equal to zero (Bańbura et al., 2010). For coefficients on lags of other variables we increase the penalty slightly by multiplying *λ* with *l*^2^ instead of (*l* − 1)^2^. 4

### 3.3 Sparsification of the variance–covariance matrix

Up to this point, we have focused attention on obtaining a sparse representation of the VAR coefficients. In large dimensions, **Σ** also contains *w* free elements and, without using more sophisticated shrinkage techniques, the existing estimate would be prone to overfitting. As a potential remedy, we propose postprocessing the estimates of the precision matrix **Σ**^−1^ (i.e., the inverse of **Σ**). Friedman, Hastie, and Tibshirani (2008) and, more recently, Bashir, Carvalho, Hahn, and Jones (2019) propose methods to ex‐post sparsify precision matrices using the graphical lasso. We follow this literature and specify a loss function similar to Equation (11) that aims to strike a balance between model fit and parsimony.

Let **Ω** be a sparse estimate of **Σ**^−1^ with elements given by *ω*_*ij*_. The loss function is then given by

(15) $${\hat{\Omega }}^{*}=\underset{\Omega }{\text{arg min}}\left\{\text{tr}\left(\Omega \hat{S}\right)-\log\left(\det(\Omega )\right)+\underset{i\neq j}{\sum }\rho_{ij}|\omega_{ij}|\right\},$$

with $\hat{S}$ denoting an estimate of the variance–covariance matrix, *ρ*_*ij*_ referring to a parameter‐specific penalty and $\log\left(\det(•)\right)$ being the log‐determinant while tr(•) denotes the trace of a square matrix. The term $\text{tr}\left(\Omega \hat{S}\right)-\log\left(\det(\Omega )\right)$ measures the (negative) expected fit whereas $\sum_{i\neq j}\rho_{ij}|\omega_{ij}|$ constitutes a penalty term that penalizes nonzero precision parameters in **Ω**. Similarly to Equation (11), Equation (15) aims to find a sparse precision matrix that describes the data well while being parsimonious. 5

Optimizing Equation (15) is challenging, and suitable penalty parameters need to be defined. We follow Friedman et al. (2008) in adopting the coordinate descent algorithm and state Equation (15) as a set of independent soft‐threshold problems that can be solved for each off‐diagonal element, respectively.

To determine the penalty parameter, we follow Friedman, Hastie, and Tibshirani (2019) and use

(16) $$\rho_{ij}=\frac{\varpi }{|{\hat{s}}_{ij}^{*}{|}^{\frac{\kappa }{2}}},$$

where $\left|{\hat{s}}_{ij}^{*}\right|$ denotes the absolute size of the (*i*, *j*)th element of ${\hat{S}}^{-1}$ and *ϖ* is a scalar penalty parameter whereas *κ* ≥ 1 controls the penalty on small precision parameters. Equation (16) nests the specification stipulated in Bashir et al. (2019) if we set κ=1, ${\hat{s}}_{ij}$ to an initial estimate of the (*i*, *j*)th element of the precision matrix, and cross‐validate *ϖ*.

It is worth discussing a promising alternative approach to regularization of precision matrices. One could also regularize **Σ**^−1^ by stating it as a set of nodewise regressions (Meinshausen & Bühlmann, 2006). Exploiting the triangular decomposition of the precision matrix one can treat each node as an independent lasso problem and use the other endogenous variables as covariates. This strategy would imply that one replaces the optimization problem in Equation (15) by a set of *m* independent (node‐specific) problems as outlined in Equation (11). As noted by Friedman et al. (2008) and Banerjee, Ghaoui, and D'Aspremont (2008), however, this approach is a special case of the graphical lasso and thus closely related.

### 3.4 Posterior inference in sparse VARs

Before discussing our posterior simulation algorithm, it is worth noting that up to this point, the different sparsification techniques have been proposed such that some estimate (i.e., the posterior mean/median) is used and then ex‐post sparsified. This technique provides a sparse point estimator of ***a*** and **Σ** but is not capable of controlling for posterior uncertainty conditional on zeroing out the *v*_*ij*_'s.

Following Huber et al. (2020), we sparsify *each draw* from the joint posterior distribution of ***a*** and **Σ**. Drawing from the joint posterior is easily achieved. Let ***a***^(*r*)^ and **Σ**^(*r*)^ denote the *r*th draw from the posterior, then we first sample **Σ**^(*r*)^ from its marginal posterior distribution in Equation (9) and, conditional on this draw, we sample ***a***^(*r*)^ from Equation (6). Given this pair of draws, the corresponding loss functions become

(17) $${\hat{a}}^{*(r)}=\underset{\alpha }{\text{arg min}}\left\{\frac{1}{2}{||\left(Za^{\left(r\right)}-Z\alpha \right)||}_{2}^{2}+\overset{k}{\underset{j=1}{\sum }}\kappa_{j}^{\left(r\right)}|\alpha_{j}|\right\},$$

(18) $${\hat{\Omega }}^{*(r)}=\underset{\Omega }{\text{arg min}}\left\{\text{tr}\left(\Omega \Sigma^{\left(r\right)}\right)-\log\left(\det(\Omega )\right)+\underset{i\neq j}{\sum }\rho_{ij}^{\left(r\right)}|\omega_{ij}|\right\}.$$

Equations (17) and (18) indicate that we search for an optimal action that minimizes the loss (instead of the expected loss) *for each draw*. This guarantees that the corresponding sparse estimates associated with the *r*th draw of ${\hat{a}}^{*(r)}$ and ${\hat{\Omega }}^{*(r)}$ are optimal. To be consistent with the definition of the (variable‐specific) penalty parameters in Equations (13) and (16), we replace the corresponding point estimators with the draws of *a*_*j*_ and *s*_*ij*_.

This approach is similar in nature to Woody, Carvalho, and Murray (2020), who performed (approximate) uncertainty quantification around sparse estimators. As opposed to our approach, Woody et al. (2020) estimated a regression model using MCMC and then project each draw into the sparse posterior for the optimal model. This is very similar to our strategy with the main exception that we base our inferences on all sparsified MCMC draws. More precisely, while Woody et al. (2020) relied on a single optimal model (selected using the posterior mean) to project the nonsparse posterior draws into the sparse regression with *q* selected covariates, our approach allows for uncertainty about this set of *q* regressors. In the simulation exercise, we show that the corresponding (sparse) point estimate is close to the one of the traditional approach and thus works well empirically.

Our approach can be viewed as an approximate algorithm to draw from the joint posterior of sparsified coefficients $p({\hat{a}}^{*},{\hat{\Omega }}^{*}|Y,X)$. The optimization problem in Equation (17) is solved using the SAVS estimator. This approach, however, has been developed under the assumption that the point estimate used is the posterior mean/median. Ray and Bhattacharya (2018) show that using any of these implies that the gradient descent algorithm converges quickly (after one iteration). Using draws from the posterior of ***a*** instead yields similar favorable properties of the optimization algorithm, leading to convergence after one iteration. 6

As opposed to the approach proposed in Hahn and Carvalho (2015), our method allows for uncertainty quantification and computation of nonlinear functions of the parameters such as impulse responses or higher‐order predictive distributions. Moreover, it allows for selecting appropriate submodels (defined through inclusion/exclusion of covariates and/or covariance relations in **Σ**). Applying sparsification to each draw implicitly yields a sparse estimator of ***a*** and **Σ**, which can be viewed as a specific restricted version of the nonsparsified model. Because we average across these different sparse estimators, we effectively average across different models. Doing this is similar to Bayesian model averaging. In contrast, the traditional method can be viewed as approximate Bayesian model selection with the shortcoming that uncertainty across models is not taken into consideration.

As mentioned in Section 1, applying the SAVS algorithm to sparsify draws from the joint posterior during MCMC potentially implies that point estimators such as the posterior mean of ${\hat{a}}^{*}$ and ${\hat{\Sigma }}^{*}$ are nonsparse. However, this strongly depends on the information contained in the posterior distribution; if there is significant information that a given coefficient is equal to zero, the corresponding point estimator of the sparsified coefficient could also be exactly zero.

## 4 SIMULATION‐BASED EVIDENCE

We use a set of different data generating processes (DGPs) that vary in terms of dimension (*m* ∈ {3, 10, 30}), length of the time series (*T* ∈ {80, 240}), and whether the model is sparse, moderately sparse, or dense to analyze if sparsification improves estimation accuracy. All simulated VAR models feature five lags ( p=5), with the coefficient matrix **A**_*j*_ (for j=1,…,5) being drawn from $N(0,{\left(\xi /j\right)}^{2})$. In the case of m=3, we set ξ=0.3, and for stability reasons, we define ξ=0.2 for m=10 and ξ=0.1 for m=30. Moreover, we add 0.25 to the diagonal elements of **A**_1_. To capture that higher lag orders become less important, we rescale the variance of the Gaussian by 1/*j*^2^ (for j=2,…,5). Similarly, the nonzero off‐diagonal elements of the lower Cholesky factor of **Σ** are sampled from $N(0,\xi^{2})$, whereas the elements of diag(**Σ**) are all nonzero and set to 0.25.

Sparsity is introduced by randomly setting off‐diagonal elements in **A**_*j*_ (for j=1,…,5) and in the lower Cholesky factor of **Σ** to zero. As stated above, we consider three levels of sparsity. The dense model features around 10*%* zeroes in the coefficients whereas the moderately sparse model features around 60*%* zeroes. Finally, we also consider an extremely sparse DGP with approximately 90*%* zeroes. The dense DGP turns out to be a challenging case for our model. This is because it features a large number of nonzero but small coefficients (especially for **A**_*j*_ with *j* > 1), which might be erroneously set equal to zero.

To assess the sensitivity of the results with respect to different choices of *λ* and *ϖ*, we compute a range of sparse models and benchmark them to the nonsparse competitor. This nonsparse competitor is a Minnesota‐prior BVAR with hyperparameters obtained by optimizing the marginal likelihood of the model over a grid (see, e.g., Carriero et al., 2019). Moreover, we consider the stochastic search variable selection (SSVS) prior as competitor (George & McCulloch, 1993, 1997). This model assumes a mixture of Gaussians prior to introduce sparsity but has the severe drawback of being nonconjugate and thus challenging to estimate in large dimensions. For the SSVS, we follow George et al. (2008) and rescale the spike and slab component with the OLS coefficient variances denoted by ${\hat{v}}_{ii}$. The corresponding spike variance is then given by $0.01\times {\hat{v}}_{ii}$, whereas the slab variance is considerably larger with $100\times {\hat{v}}_{ii}$.

Finally, we add two additional competing specifications. The first one (labeled SAVS‐Median) is the traditional SAVS approach stipulated in Ray and Bhattacharya (2018), which sparsifies the posterior median. This model allows us to assess whether sparsifying the posterior median yields similar insights (in terms of point estimates) as our approach. The second one, labeled CDA, sparsifies each draw from the joint posterior using a standard coordinate descent algorithm (i.e., without stopping after the first iteration). This specification serves to illustrate whether using more iterations yields similar insights compared with stopping after the first iteration.

Table 1 shows (relative) mean absolute errors (MAE) between the posterior median of the coefficients for the sparsified BVAR and the true parameter values, averaged across 150 replications per DGP. Note that all numbers in the table feature a numerical standard error which is relatively small. Nevertheless, these findings need to be interpreted with some caution and we aim to focus on results that imply substantial differences relative to the benchmark after taking into account the simulation‐induced variation. All MAEs are divided by the MAEs of the nonsparse competitor.

**TABLE 1:** MAE ratios of coefficients and covariances to nonsparse BVAR estimates

	DGP				Specification						Alternatives with λ=1	
	*m*	*T*	Sparsity		MIN ‐ λ=0.01	MIN ‐ λ=0.1	MIN ‐ λ=0.5	MIN ‐ λ=1	SSVS		SAVS ‐ Median	CDA
	Coefficients			
	S	80	Sparse		0.654	0.504	0.451	**0.446**	0.546		0.446	0.446
			Moderate		0.727	0.636	**0.623**	0.629	0.669		0.629	0.629
			Dense		0.847	**0.818**	0.830	0.846	0.860		0.846	0.846
		240	Sparse		0.618	0.454	0.414	**0.413**	0.505		0.413	0.413
			Moderate		0.717	0.612	**0.604**	0.611	0.651		0.611	0.611
			Dense		0.844	**0.819**	0.846	0.865	0.865		0.865	0.865
	M	80	Sparse		0.637	0.491	0.436	**0.436**	0.632		0.436	0.436
			Moderate		0.758	0.697	**0.696**	0.710	0.797		0.710	0.710
			Dense		**0.913**	0.924	0.970	0.999	1.018		0.999	0.999
		240	Sparse		0.588	0.423	**0.377**	0.382	0.505		0.382	0.382
			Moderate		0.716	**0.642**	0.644	0.665	0.692		0.665	0.665
			Dense		**0.886**	0.890	0.927	0.960	0.963		0.960	0.960
	L	80	Sparse		0.579	0.507	**0.507**	0.507	1.572		0.507	0.507
			Moderate		0.800	**0.794**	0.798	0.798	1.513		0.798	0.798
			Dense		**0.984**	1.023	1.032	1.032	1.432		1.032	1.032
		240	Sparse		0.577	0.441	**0.439**	0.442	0.704		0.442	0.442
			Moderate		0.760	**0.735**	0.770	0.779	0.876		0.779	0.779
			Dense		**0.953**	1.005	1.070	1.086	1.075		1.086	1.086
	Covariances			
	S	80	Sparse		0.996	0.971	0.903	0.847	**0.647**		0.847	0.847
			Moderate		0.998	0.981	0.935	0.899	**0.792**		0.899	0.899
			Dense		1.000	**1.000**	1.001	1.007	1.090		1.007	1.007
		240	Sparse		0.987	0.919	0.771	**0.694**	0.700		0.694	0.694
			Moderate		0.994	0.955	0.871	0.835	**0.829**		0.835	0.835
			Dense		0.999	**0.999**	1.010	1.039	1.153		1.039	1.039
	M	80	Sparse		0.998	0.980	0.918	0.859	**0.594**		0.859	0.859
			Moderate		0.999	0.988	0.953	0.922	**0.826**		0.922	0.922
			Dense		1.000	**0.999**	1.000	1.007	1.174		1.007	1.007
		240	Sparse		0.992	0.936	0.788	0.687	**0.639**		0.687	0.686
			Moderate		0.996	0.973	0.915	0.889	**0.863**		0.889	0.889
			Dense		**1.000**	1.003	1.032	1.081	1.248		1.081	1.082
	L	80	Sparse		0.998	0.985	0.934	0.882	**0.632**		0.882	0.881
			Moderate		0.999	0.988	0.950	0.913	**0.809**		0.913	0.912
			Dense		0.999	0.992	0.968	**0.950**	1.004		0.950	0.950
		240	Sparse		0.993	0.938	0.780	0.658	**0.604**		0.658	0.656
			Moderate		0.996	0.966	0.887	0.841	**0.827**		0.841	0.840
			Dense		0.999	**0.993**	0.998	1.026	1.106		1.026	1.026

*Note*: Bold numbers indicate the smallest MAE ratios. We simulate a DGP for a *small‐scale*( m=3), *medium‐scale* ( m=10), and *large‐scale* ( m=30) VAR for two different number of observations *T* and for three different degrees of sparsity (zero parameters as percentage of total number of coefficients k=m(mp+1) and covariances w=m(m+1)/2, ranging from a dense DGP to a fully sparse DGP. SAVS‐Med. refers to a sparse estimator, where we minimize the expected loss of the posterior median (see Hahn & Carvalho, 2015), whereas for the CDA specification, we replace the SAVS estimator with a coordinate descent algorithm; that is, we do not stop after the first iteration for both coefficients and covariances.

The upper panel of Table 1 presents the results for the VAR coefficients, whereas the lower panel displays the MAEs associated with the covariance parameters. In order to investigate how differing values of *λ* and *ϖ* impact estimation accuracy, we also estimate the model over a grid of values for *λ* ∈ {0.01, 0.1, 0.5, 1} and set ϖ=λ/10. Before proceeding, it is worth noting that we estimate all VAR models with five lags.

Considering the upper panel of Table 1, a few results are worth emphasizing. First, we observe that sparsification pays off in terms of achieving lower estimation errors. These improvements rise with the true level of sparsity and decrease with the length of the sample. Especially when *T* is small relative to the number of parameters, sparsification improves against the traditional Bayesian VAR model.

Second, the sensitivity of estimation accuracy with respect to *λ* varies with the level of sparsity. For instance, we find slightly more pronounced differences if the DGP is either dense or moderately dense, but as long as *λ* is set greater to 0.01, we find only small differences in MAEs. It is worth noting, however, that these small differences across different penalty terms are often insignificant. Once we take into account numerical standard errors, the specific choice of *λ* (as long as it is not set too small) seems to play a minor role with differences being smaller than 10% in MAE terms (and thus often within one standard deviation). This also provides some evidence that the specific choice proposed in Ray and Bhattacharya (2018) (i.e., λ=1) works well in most circumstances.

Third, for large models, we find that the SAVS estimator yields substantial gains, improving upon the shrinkage‐only estimator by large margins. These improvements even arise if the DGP is characterized by relatively few zeros in the VAR coefficients. This finding is not surprising given the fact that the absolute number of zeros increases with the dimension of the parameter space and the small but negligible posterior estimates under the Minnesota BVAR have a detrimental effect on estimation accuracy.

Fourth, we find that the conjugate VARs in combination with SAVS very often improve upon the nonconjugate and more flexible VAR coupled with the SSVS prior. For large‐scale models, we even find that the SSVS prior performs rather poorly and this might be caused by mixing issues in the indicators that determine which of the Gaussians is chosen. In smaller‐sized models, accuracy differences decrease but still favor our proposed sparsified model.

Finally, comparing the performance of the SAVS‐Median and CDA approaches with the corresponding model based on setting λ=1 reveals no differences in estimation accuracy. This remarkable result shows that sparsifying each draw from the joint posterior yields (almost) identical sparsified point estimators and provides evidence that using only a single iteration of the coordinate descent algorithm seems to be sufficient when compared with using a stopping rule (and thus potentially many more iterations).

The lower panel of Table 1 provides similar but more mixed insights. Sparsification of the variance–covariance matrix sometimes yields accuracy improvements over its nonsparsified counterpart. These improvements range from being small (or in some rare cases even negative) to very large (in case the DGP is sparse and the model is moderately large).

Considering the performance of the SSVS prior shows that it provides more accurate estimates of **Σ** but at substantially higher computational costs. In most instances where the SSVS prior improves upon our SAVS‐based model, these performance gains are often small (i.e., below 10% to 15% in MAE terms). So given that the additional costs of applying SAVS to the posterior draws of **Σ** are small (see Table S3), we can recommend adding this additional step to further improve estimation accuracy.

**FIGURE 1:**
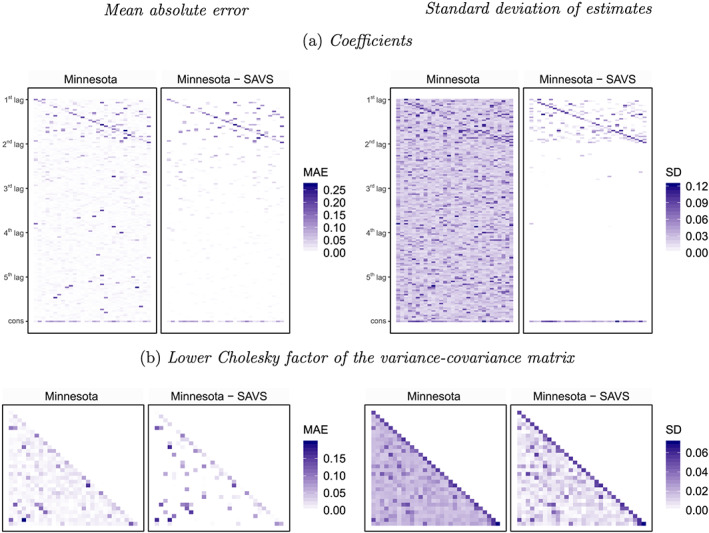
Heatmaps of coefficients and covariances for m=30 endogenous variables, p=5, T=240, and the degree of sparsity is 90*%*. *Notes*: Panel (a) lists the *m* endogenous variables on the *x*‐axis and the (*mp*+1) regressors for each equation on the *y*‐axis. The *i*^*th*^ (*m*×*m*) block denotes the *i*^*th*^ lag coefficient matrix, while the constant is ordered last (indicated by cons). Moreover, in panel (b) the lower Cholesky factor of the variance‐covariance matrix is an (*m*×*m*)‐dimensional lower triangular matrix [Colour figure can be viewed at wileyonlinelibrary.com]

We stressed one key advantage in Section 3, namely, that sparsification reduces estimation uncertainty by zeroing out the coefficient under scrutiny during posterior simulation. Thus, while the discussion in the previous paragraphs highlights that using sparsification improves estimation performance in terms of point estimators, we now investigate its consequences on the posterior variance of ***a***. Figure 1a and Figure 1b are heatmaps that show the absolute distance between the posterior median and the true coefficients (left panel) as well as a corresponding heatmap that presents the posterior standard deviation of the parameter under scrutiny (right panel). These heatmaps are created for a single realization from the sparse DGP with T=240 and m=30.

Considering the heatmaps reveals that sparsification improves estimation accuracy and accurately detects zeroes, as evidenced by the abundance of white cells in the figure. The slight bias along the main diagonal (which also exists under the shrinkage‐only model) stems from the informative prior that is centered around zero. However, note that even with a high degree of shrinkage, the corresponding estimate of ***a*** with the Minnesota prior is quite dense. By contrast, applying SAVS yields a very sparse coefficient matrix and, in addition, a sparsified estimate of the variance–covariance specification.

The left panel of Figure 1 shows that sparsity is, not surprisingly, accompanied by appreciable decreases in posterior variance. White cells imply that the posterior standard deviation is (close to) zero. This is often the case if we postprocess the posterior using SAVS. As expected, we see that the coefficients associated with the first lag often feature considerable posterior uncertainty. For higher lag orders, however, SAVS effectively reduces posterior uncertainty and, in light of the strong accuracy gains for the point estimator, leads to a much more favorable bias–variance relationship. For a standard Minnesota prior without SAVS, we observe a more dense heatmap with a great deal of purple shaded areas. We would like to emphasize that even for the Minnesota prior, these standard deviations are often small (especially compared with some weakly informative prior). But these small elements in *v*_*ij*_ could add up (see Equation 10) and thus be deleterious for predictive accuracy. And it is precisely this problem that we try to circumvent by applying SAVS.

On the variance–covariance matrix, we again see more white cells under the sparse model. This shows that we reduce estimation uncertainty. Considering the right panel of Figure 1b, moreover, implies that we also successfully reduce posterior uncertainty around the free elements in **Σ**.

## 5 FORECASTING APPLICATION

### 5.1 Data overview, design of the forecasting exercise, and competitors

To illustrate the merits of our approach for forecasters in central banks and other policy institutions, we now carry out a U.S. macroeconomic forecasting exercise. Several recent papers have analyzed this dataset using various shrinkage and sparsification techniques (Cross, Hou, & Poon, 2020; Giannone, Lenza, & Primiceri, 2017) and find mixed evidence for sparsity. Nevertheless, using this application, we aim to illustrate that even when there is little evidence in favor of sparsity, the proposed framework is still capable of improving upon a model that relies solely on shrinkage priors.

In this application, we use the quarterly variant of the McCracken and Ng (2016) dataset that spans the period from 1959:Q1 up to 2018:Q4. To investigate whether combining shrinkage and sparsity improves predictive performance, we rely on a recursive forecasting design. Using the period that runs from 1959:Q1 to 1989:Q4 as an initial training period to compute the *h*‐step‐ahead predictive distribution (for *h* ∈ {1, 4, 8}), we expand the initial estimation period by one quarter and repeat this procedure until we reach the penultimate point in the sample (i.e., 2018:Q3). The period from 1990:Q1 to 2018:Q4 serves as a hold‐out period to evaluate the predictive accuracy of the models using root‐mean‐squared forecast errors (RMSEs), log predictive likelihoods (LPLs), and normalized forecast errors. 7

The existing literature highlights the necessity to exploit large information sets (see, for instance, Bańbura et al., 2010; Carriero, Clark, & Marcellino, 2015; Giannone et al., 2015; Koop, 2013). Building on this evidence, we apply our techniques to a VAR model that features m=165 macroeconomic and financial variables. Out of these, we select three traditional target variables: output (GDPC1), consumer price inflation (CPIAUCSL), and the Federal Funds Rate (FEDFUNDS). Because the computational burden of using nonconjugate priors increases dramatically with model size, we do not use the SSVS prior for the large dataset.

Apart from this large‐scale VAR (L‐VAR), we investigate how our techniques perform across different model sizes and dimension‐reduction techniques. These competing models range from small‐ and medium‐scale VARs to dynamic factor models in the spirit of Bernanke, Boivin, and Eliasz (2005). These competing approaches are as follows 8:

- **S‐VAR:** This specification is inspired by the literature using small‐scale three equation VAR models that feature the three target variables exclusively.
- **M‐VAR:** This model extends the small‐scale information set by additionally including financial market variables. In total, this specification includes m=21 variables and thus resembles the size of typical reduced‐form models employed by the ECB to carry out its short‐term inflation projections.
- **FA‐VAR:** As a competitor that exploits the full information set but reduces the dimensionality of the estimation problem, we use a factor‐augmented VAR (FA‐VAR). This model augments the small‐scale VAR by including three principal components extracted from the remaining quantities (see Bańbura et al., 2010; Koop, 2013).

All models feature p=5 lags of the endogenous variables.

### 5.2 Choice of hyperparameters

Because we use VAR models that do not only differ in the size of their information sets but also how these information is used during estimation, careful choice of the prior hyperparameters is necessary. Using the prior outlined above, we need to set *θ*_1_ as well as the sparsification parameters *λ* and *ϖ*.

To set the hyperparameters of the Minnesota prior, we follow two different routes. The first (and simplest) way is to set *θ*_1_ such that shrinkage increases with the size of the information set (see, for instance, Bańbura et al., 2010; Koop, 2013) and select *θ*_1_ over a grid of potential values.

The second approach is based on optimizing the marginal likelihood (which is available in closed form) a priori (see Carriero et al., 2015). One problem with this strategy, however, is that the marginal likelihood might be ill‐behaved, which renders optimization difficult. 9

Optimizing the marginal likelihood in huge models is often unfeasible due to numerical reasons. As a solution, we assess the sensitivity of the forecasts with respect to three choices of the hyperparameter $\theta_{1}=\{0.025,0.05,0.075\}$. These three values all lead to an informative prior, but the weight placed on prior information ranges from being large ( $\theta_{1}=0.025$) to moderate ( $\theta_{1}=0.075$). Using these values enables us to assess how shrinkage and sparsification interact. For example, if *θ*_1_is set to 0.025, it is very likely that the SAVS estimator will lead to a sparse model while a shrinkage parameter $\theta_{1}=0.075$ allows for larger elements in **a** and thus a more dense model under the SAVS estimator.

For the small‐ and medium‐scale models and the FA‐VAR specification, we define a large grid of values for *θ*_1_ ∈ {0.01, 0.025, 0.050, 0.075, 0.10, 0.125, 0.15, 0.20, 0.25, 0.30, 0.35, 0.40, 0.45, 0.50, 0.75, 1, 2, 5}. Using this grid, we seek the value of *θ*_1_ that maximizes the marginal likelihood. Over the hold‐out sample, this procedure yields an average of $\theta_{1}=0.457$ for the small‐scale model, $\theta_{1}=0.392$ for the FA‐VAR specification, and $\theta_{1}=0.149$ for the medium‐scale model. These values indicate that the larger the model becomes, the more weight needs to be placed on the prior. Similar to Carriero et al. (2015), we find that the hyperparameters tend to display little variation over the hold‐out period. For example, in the case of the medium‐scale model, we find that *θ*_1_ ranges from 0.125 to 0.15.

Finally, we investigate how forecasting performance changes for different values of *λ* and *ϖ*, again, using a grid of candidate values. More precisely, we set *λ* ∈ {0.01, 0.1, 0.5, 1} and ϖ=λ/10.

### 5.3 Point forecasting performance

In this subsection, we first consider point forecasting accuracy of the different models and compare sparse with nonsparse models. Table 2 depicts the relative RMSEs to a small‐scale VAR with a Minnesota prior (henceforth called the benchmark model) and without sparsification for the three target variables. The asterisks indicate statistical significance for each model relative to the benchmark as measured by the Diebold and Mariano (1995) test. The numbers in parentheses refer to the ranking of the three best specifications using the procedure outlined in Hansen, Lunde, and Nason (2011).

**TABLE 2:** RMSE ratios relative to the *small‐scale* BVAR with a Minnesota prior

	Specification		Average				Marginal one‐quarter‐ahead				Marginal one‐year‐ahead				Marginal two‐year‐ahead		
	Specification		One‐q.‐ahead	One‐y.‐ahead	Two‐y.‐ahead		GDPC1	CPIAUCSL	FEDFUNDS		GDPC1	CPIAUCSL	FEDFUNDS		GDPC1	CPIAUCSL	FEDFUNDS
	**S‐VAR**	
	MIN ‐ λ=0.01		0.980 (3)	1.003	1.000		1.008	**0.975 (1)**	0.951		1.008	0.996	1.049		1.010	0.994	1.024
	MIN ‐ λ=0.1		0.977 (2)	0.999	0.994		1.002	0.992	0.833		1.021	0.995	0.973		0.979	0.994***	1.039
	MIN ‐ λ=0.5		**0.976 (1)**	1.000	0.997		1.005	1.000	0.756** (3)		1.002	1.000	0.995		1.015	0.984	1.077
	MIN ‐ λ=1		0.981	0.996	0.982		1.004	1.011	0.736*** (2)		0.996	0.998	0.983		0.984	0.977	1.033
	SSVS		0.991	1.030	0.987		1.018	0.989 (2)	0.948		1.059	1.021	1.029		1.017	0.975	1.017
	**FA‐VAR**	
	MIN		1.041	1.031**	1.006		0.937	1.022*	1.317		1.083***	1.020	0.977		1.083	0.987	**0.943 (1)**
	MIN ‐ λ=0.01		1.024	1.013	1.008		0.905	1.015	1.272		1.043	1.010	0.959		1.020	1.000	1.053
	MIN ‐ λ=0.1		1.025	1.019	0.990		0.903	1.037	1.185		1.081**	0.996	1.042		0.999	0.983	1.034
	MIN ‐ λ=0.5		1.047	1.027	1.001		0.909	1.066*	1.201		1.110**	0.993*	1.095		1.001	0.995	1.066
	MIN ‐ λ=1		1.064	1.014	0.995		0.903	1.092*	1.203		1.055	1.000	1.017		0.980	0.992**	1.072
	SSVS		1.164***	1.181***	1.154***		0.857	1.213***	1.411***		1.116***	1.198***	1.204**		1.074*	1.161***	1.317***
	**M‐VAR**	
	MIN		1.067	0.985	1.006		0.844 (3)	1.112**	1.216		1.011	0.980 (2)	0.960*		1.039	0.996	1.010
	MIN ‐ λ=0.01		1.041	0.983 (2)	0.997		0.815** (1)	1.114**	1.040		0.996	0.982 (3)	0.953		1.007	0.993	1.011
	MIN ‐ λ=0.1		1.011	1.008	0.985		0.837*** (2)	1.098**	0.817***		1.020	1.004	1.012		1.010	0.972 (2)	1.051
	MIN ‐ λ=0.5		1.002	0.999	**0.972 (1)**		0.939	1.065	0.725*** (1)		1.011	0.995	0.994		0.978	**0.964 (1)**	1.027
	MIN ‐ λ=1		1.025	0.993	0.988		1.013	1.070	0.759**		1.004	0.992	0.975		0.995	0.981	1.037
	SSVS		1.189***	1.223***	1.215***		0.938	1.240***	1.353***		1.147*	1.216***	1.472***		1.129	1.212***	1.490***
	**L‐VAR** ( $\theta_{1}=0.025$)	
	MIN		1.000	**0.978 (1)**	0.995		0.854**	1.068	0.876		0.994 (3)	**0.975 (1)**	0.956		1.029	0.983	1.010 (3)
	MIN ‐ λ=0.01		1.041	0.994	0.983		0.996	1.096*	0.782**		1.020	0.989	0.963		0.990	0.978	1.023
	MIN ‐ λ=0.1		1.055	1.003	0.991		1.005	1.105*	0.848**		1.020	1.002	0.966		0.988	0.986	1.061
	MIN ‐ λ=0.5		1.045	0.990	0.983		1.021	1.087	0.833*		0.992 (2)	0.992	0.967		0.969 (2)	0.982	1.035
	MIN ‐ λ=1		1.046	0.999	0.980		1.025	1.085*	0.851*		1.010	1.001	0.947 (2)		0.966* (1)	0.980	1.026
	**L‐VAR** ( $\theta_{1}=0.05$)	
	MIN		1.023	1.000	0.984 (3)		0.881	1.055	1.110		1.010	1.000	0.970		1.011	0.976	0.974 (2)
	MIN ‐ λ=0.01		1.020	0.994	0.983		0.891*	1.091	0.848**		0.995	0.995	0.975		1.007	0.972 (3)	1.023
	MIN ‐ λ=0.1		1.039	0.995	0.984		0.986	1.092*	0.823**		1.023	0.987	0.992		0.982	0.982	1.011
	MIN ‐ λ=0.5		1.052	1.003	0.983* (2)		1.032	1.091	0.854*		1.006	1.003	0.987		0.981*	0.981	1.013
	MIN ‐ λ=1		1.043	0.993	0.986		1.020	1.084*	0.837*		0.998	0.993	0.978		0.990	0.980	1.035
	**L‐VAR** ( $\theta_{1}=0.075$)	
	MIN		1.037	0.997	1.011**		0.921	1.042	1.222		1.004	0.997	0.983		1.059*	0.995	1.023
	MIN ‐ λ=0.01		1.039	0.984 (3)	0.984		0.885	1.104*	0.963		**0.982 (1)**	0.988	0.961		0.973* (3)	0.983	1.036
	MIN ‐ λ=0.1		1.029	0.993	0.985		0.981	1.084*	0.780**		1.003	0.996	**0.943 (1)**		0.984	0.979	1.042
	MIN ‐ λ=0.5		1.050	0.994	0.984		1.056	1.087	0.799**		1.004	0.996	0.949* (3)		0.993	0.980	0.996
	MIN ‐ λ=1		1.050	0.993	0.990		1.036	1.094*	0.806**		1.016	0.989	0.957		0.990	0.988	1.016

*Note*: Numbers smaller than one imply that a given model improves upon the benchmark, whereas values greater than one suggest that the benchmark yields more precise point predictions. Bold numbers indicate lowest RMSE ratios for each horizon and target variable (and therefore best performing models over the full hold‐out sample), while the numbers in parenthesis refer to the ranking of the three best specifications according to the MCS (Hansen et al., 2011) procedure (including the benchmark). Gray shaded rows denote nonsparsified models.

^∗∗∗^Statistical significance for each model relative to the benchmark at the 1% significance level.

^∗∗^Statistical significance for each model relative to the benchmark at the 5% significance level.

^∗^Statistical significance for each model relative to the benchmark at the 10% significance level.

We start by considering the average model performance (in terms of computing average RMSEs across the three focus variables) for the one‐step‐ahead forecasts. In general, there exists no single superior modeling approach that outperforms its competitors in a statistically significant manner. However, when it comes to lowest RMSEs, our results suggest that the most accurate one‐step‐ahead forecasts can be found for smaller‐sized models with sparsification (with the S‐VAR with λ=0.5 yielding the lowest average RMSEs). Irrespective of *λ*, sparse small‐scale models perform well and are the only ones that improve upon the benchmark Minnesota BVAR.

To analyze point forecasts in more detail, we now discuss the one‐step‐ahead forecasting performance across the three focus variables. For output, we observe that most large models display relative RMSEs below or close to one, suggesting that their predictions tend to improve forecast accuracy compared with the ones obtained from using the simple small‐scale Bayesian VAR. When we compare sparsified large‐scale BVARs with the nonsparsified counterparts, we find limited evidence that sparsification improves point forecasts (but it also never substantially hurts predictive accuracy). Turning to the medium‐scale models, we find more pronounced improvements relative to the benchmark model and that using SAVS improves predictive accuracy. These performance gains depend on the specific value of *λ*. For example, setting λ=0.01 yields a model that improves upon all competitors and produces statistically significant better forecasts than the nonsparsified benchmark model (at the five percent significance level).

Considering the one‐step‐ahead forecasts of inflation, it appears that none of the larger‐scale competitors is capable of improving upon the benchmark small‐scale VAR. Notice, however, that for the medium‐sized model and the FA‐VAR specification, introducing sparsity through our SAVS estimator sometimes yields more accurate short‐run predictions and tends to outperform not only the nonsparsified model but also the SSVS prior. In general, and similarly to output, we find relative RMSEs close to unity (and in fact often slightly exceeding unity).

Analyzing one‐quarter‐ahead interest rate forecasts reveals that medium‐ to large‐sized models yield forecasts that are more accurate than the ones obtained from the benchmark VAR. This result is particularly pronounced for the moderately‐sized VAR and λ=0.5. Moreover, using SAVS in combination with shrinkage improves accuracy markedly. These improvements are substantial and often statistically significant.

Next, we consider four‐ and eight‐step‐ahead predictions. The results differ from the ones discussed above. While we find again mixed evidence that sparsification improves point predictions, one striking difference between one‐ and multi‐step‐ahead forecasts is that more information seems to be beneficial for accurately forecasting multiple periods ahead. For the one‐year‐ahead horizon, our findings suggest that a nonsparsified L‐VAR with a tight Minnesota prior ( $\theta_{1}=0.025$) yields the most accurate point forecasts. For 2‐year‐ahead forecasts, the sparse medium‐scale model (with λ=0.5) dominates all competitors.

In terms of the three focus variables, we find small gains for the larger models when interest focus on predicting output 1‐year‐ahead. When 2‐year‐ahead output predictions are considered, large VARs with sparsification work well and improve upon the nonsparse models. For 1‐year‐ahead inflation forecasts, we find limited evidence that SAVS improves forecast accuracy. However, adding the SAVS step slightly improves 2‐year‐ahead inflation forecasts for some of the larger VARs. In terms of interest rate forecasts, we still find that SAVS improves predictive accuracy but these improvements diminish with higher order forecasts.

Overall, there is only mixed evidence that point forecasts can be improved by adding the SAVS step. There are cases where gains from SAVS become more pronounced (such as longer run forecasts for output and inflation or short‐term interest rate forecasts), but there also exist several instances where using a sparse model slightly hurts forecast accuracy. One important thing to note is that sparsification only very rarely harms predictive accuracy in a statistically significant manner.

To further substantiate this observation we consider the model confidence set (MCS) procedure of Hansen et al. (2011), implemented by Bernardi and Catania (2018), to obtain a measure for *model uncertainty*. Table 3 shows the cardinality of the superior model set across variables and forecast horizons. Moreover, the table shows the number of sparsified models included in the MCS. Considering a total number of 33 models (including the benchmark) and using a mean squared error loss function, the MCS procedure suggests a quite large superior model set, including around 26 to 33 models. These numbers strongly depend on the variable and forecast horizon considered. In general, only few specifications are eliminated by the MCS procedure with no single class of models significantly outperforming its alternatives. It is worth emphasizing that in most instances, the share of models using SAVS is high.

**TABLE 3:** Number of models included in the superior models sets (SMSs) for point forecasts according to different shrink‐and‐sparsify specifications

			Average				Marginal one‐quarter‐ahead				Marginal one‐year‐ahead				Marginal two‐year‐ahead		
			One‐q.‐ahead	One‐y.‐ahead	Two‐y.‐ahead		GDPC1	CPIAUCSL	FEDFUNDS		GDPC1	CPIAUCSL	FEDFUNDS		GDPC1	CPIAUCSL	FEDFUNDS
	MIN		6	6	6		5	6	5		6	6	6		5	6	6
	MIN ‐ λ=0.01		6	6	6		4	6	5		6	6	5		6	6	6
	MIN ‐ λ=0.1		5	6	6		6	5	4		6	6	6		6	6	5
	MIN ‐ λ=0.5		6	6	4		5	6	6		6	6	6		6	4	5
	MIN ‐ λ=1		6	6	5		6	6	6		6	6	6		6	4	5
	SSVS		3	1	3		3	3	0		1	3	3		3	3	3
	Total		32	31	30		29	32	26		31	33	32		32	29	30

*Note*: The SMSs are obtained with the model confidence set (MCS) procedure (Hansen et al., 2011) at a 25% significance level across variables and forecast horizons. The loss function is specified in terms of mean squared errors. For each variable‐horizon combination, we use 33 different specifications (including the benchmark). Note that the SSVS prior is only considered for the three smallest information sets.

Focusing exclusively on point forecasts ignores what can be considered the main advantage of SAVS: the corresponding reduction in estimation uncertainty and the potentially positive effect on the full predictive distribution. This theme will be the subject of the next subsection.

### 5.4 Density forecasting performance

Table 4 shows differences in LPLs relative to the small‐scale Minnesota VAR, corresponding to log predictive Bayes factors (henceforth labeled BFs). Apart from focusing on variable‐specific relative LPLs, the table also displays joint BFs over the three target variables. These serve as a general measure of how well some approach performs in forecasting output, inflation and interest rates jointly. Numbers greater than zero imply that a given model improves upon the benchmark while negative BFs suggest that the benchmark yields more precise density predictions. Moreover, we compute a two‐sided Amisano and Giacomini (2007) test for each specification with a null hypothesis of equal average LPLs relative to the benchmark and report the statistical significance based on the obtained *p* values. 10We start with the joint predictive BFs and the one‐step‐ahead horizon shown in Table 4. It is worth noting that these numbers can be interpreted as a relative training sample marginal likelihood (Geweke & Amisano, 2010). For this measure, we find that the best performing model is the moderately‐sized sparse VAR with λ=0.1, suggesting that using SAVS appreciably improves density predictions.

**TABLE 4:** Log predictive likelihoods relative to the *small‐scale* BVAR with a Minnesota prior

	Specification		Joint				Marginal one‐quarter‐ahead				Marginal one‐year‐ahead				Marginal two‐year‐ahead		
			One‐q.‐ahead	One‐y.‐ahead	Two‐y.‐ahead		GDPC1	CPIAUCSL	FEDFUNDS		GDPC1	CPIAUCSL	FEDFUNDS		GDPC1	CPIAUCSL	FEDFUNDS
	**S‐VAR**	
	MIN ‐ λ=0.01		5.745**	4.828*	4.513		0.634	0.897	3.187***		1.570	0.011	1.697*		2.596***	−1.680	2.722***
	MIN ‐ λ=0.1		7.534	7.745*	6.118		0.212	0.198	6.525**		2.945	−0.850	3.558*		4.365***	−3.968	5.467***
	MIN ‐ λ=0.5		6.817	9.475	5.437		0.526	−2.596	9.088***		4.457*	−1.418	6.169***		5.880***	−7.634	8.116***
	MIN ‐ λ=1		5.288	10.737	5.783		1.515	−4.985	9.801***		5.147*	−1.903	7.587***		6.561***	−8.251	9.464***
	SSVS		−3.705	−0.176	−4.098		−3.035***	4.484 (2)	−2.205		−4.165***	**6.517 (1)**	−2.751***		−3.609***	**4.654 (1)**	−4.478***
	**FA‐VAR**	
	MIN		17.948 (3)	2.769	−3.098		11.408**	4.423 (3)	6.088		−1.815	0.329	1.871***		−3.025	−0.454	0.819
	MIN ‐ λ=0.01		19.086	5.844	2.745		11.634**	**4.606 (1)**	7.538		−0.776	1.619 (3)	2.700***		1.460	−2.279	2.561***
	MIN ‐ λ=0.1		17.368	5.394	4.983		11.687**	1.624	9.088		−1.320	0.361	2.997*		4.656***	−5.399	4.700***
	MIN ‐ λ=0.5		10.393	6.816	7.252		11.893**	−5.325	11.055*		0.072	−1.207	3.496*		6.809***	−3.661	6.073***
	MIN ‐ λ=1		5.528	10.154*	8.667		12.101**	−7.035	11.863**		1.778	−1.433	4.315**		7.417**	−3.304	6.539***
	SSVS		−46.196	−28.238	−35.739		9.724	−51.723*	−6.802		−5.828**	−30.101	−6.738**		−5.940***	−31.360	−10.958***
	**M‐VAR**	
	MIN		20.032	7.907	−1.659***		17.376**	−2.255	6.753**		0.828	5.649 (2)	−0.515		−0.931*	0.515 (3)	−0.295
	MIN ‐ λ=0.01		26.850 (2)	12.099**	8.382		20.002*** (1)	−3.443	12.838***		5.318**	0.214	6.416***		6.115**	−4.446	8.134***
	MIN ‐ λ=0.1		30.006** (1)	23.425*** (2)	13.825		19.219*** (2)	−6.633	18.867***		8.621	−3.174	14.480***		9.698*	−10.551	16.545***
	MIN ‐ λ=0.5		16.531	22.625 (3)	**18.330 (1)**		11.862***	−13.195	20.096***		10.468	−6.613	21.016***		11.235	−15.732	22.923***
	MIN ‐ λ=1		10.911	**23.489 (1)**	17.980 (2)		6.597*	−15.470	19.296***		10.689	−8.354	22.812***		11.501	−18.285	24.700***
	SSVS		−68.116	−50.493*	−67.513*		2.288	−68.574*	−3.879		−9.995**	−40.090	−7.634		−17.749**	−37.498	−19.086**
	**L‐VAR** ( $\theta_{1}=0.025$)	
	MIN		7.597	8.680	1.966		10.798**	−4.707	3.624		4.307*	0.243	3.658***		2.764*	−4.571	5.158***
	MIN ‐ λ=0.01		−1.374	16.156	14.614		5.550**	−15.415	9.195***		9.270*	−8.006	14.482***		10.447*	−11.916	16.407***
	MIN ‐ λ=0.1		−8.248	16.134	15.324		0.544	−17.598	8.403**		9.459	−9.092	15.961***		10.729*	−13.387	17.765***
	MIN ‐ λ=0.5		−6.784	16.336	15.528		0.900	−17.445	8.692**		9.528	−9.174	16.198***		10.796*	−13.520	17.978***
	MIN ‐ λ=1		−6.225	16.389	15.621 (3)		1.002	−17.345	8.827**		9.537	−9.180	16.253***		10.807*	−13.522	18.028***
	**L‐VAR** ( $\theta_{1}=0.05$)	
	MIN		6.284	5.536	−0.471		11.666**	−8.084	6.990*		3.225	0.217	2.864***		−0.132	−1.901	4.406***
	MIN ‐ λ=0.01		8.616	14.644	9.218		13.873***	−19.797*	19.791***		11.511	−18.799	23.511***		12.242	−24.021	25.737***
	MIN ‐ λ=0.1		−8.814	1.967	1.320		4.464	−33.086**	22.504***		10.734 (3)	−34.528	30.543***		12.070 (3)	−37.301	32.828***
	MIN ‐ λ=0.5		−14.061	2.130	−0.703		1.067	−38.927*	22.677***		10.718	−38.134	31.721***		12.100	−39.940	33.905***
	MIN ‐ λ=1		−12.870	2.495	−0.578		1.209	−39.186*	23.364***		10.841 (2)	−38.322	31.969***		12.144 (2)	−40.097	34.082***
	**L‐VAR** ( $\theta_{1}=0.075$)	
	MIN		6.470	2.303	−5.543		11.749*	−9.162	11.388***		2.937	0.124	0.444		−2.815	−2.295	1.106
	MIN ‐ λ=0.01		−1.536	8.305	6.836		17.102*** (3)	−28.712*	24.434***		12.856* (1)	−30.720	28.291***		**12.898 (1)**	−31.633	31.334***
	MIN ‐ λ=0.1		−4.866	−20.495	−22.643		9.246**	−40.233**	31.976*** (2)		8.744	−66.632	39.748***		10.139	−69.578	42.767***
	MIN ‐ λ=0.5		−42.144	−30.466	−30.732		−1.292	−69.624*	31.766*** (3)		7.426	−76.417	42.522***		8.980	−83.052	45.338***
	MIN ‐ λ=1		−46.109	−30.932	−31.000		−2.662	−71.845*	32.671*** (1)		7.565	−77.508	43.035*** (1)		8.984	−84.153	45.706*** (1)

*Note*: Numbers greater than zero imply that a given model improves upon the benchmark, whereas negative values suggest that the benchmark yields more precise density predictions. Bold numbers indicate highest Bayes factors for each horizon and target variable (and therefore best performing models over the full hold‐out sample in terms of density forecasts), whereas the numbers in parenthesis refer to the ranking of the three best specifications according to the MCS (Hansen et al., 2011) procedure (including the benchmark). Note for 1‐year‐ and 2‐year‐ahead FEDFUNDS density forecasts, the superior model set consists of a single best model. Gray shaded rows denote nonsparsified models.

^∗∗∗^Statistical significance for each model relative to the benchmark at the 1% significance level.

^∗∗^Statistical significance for each model relative to the benchmark at the 5% significance level.

^∗^Statistical significance for each model relative to the benchmark at the 10% significance level.

The large VARs are generally outperformed by smaller‐sized ones according to one‐step‐ahead joint BFs. For large models, we also find that sparsification hurts density forecast performance. In contrast, in the case of the small‐ and medium‐scale VARs, we find statistical significant accuracy gains from using the additional SAVS step. This pattern can also be found for the FA‐VAR.

At the one‐step‐ahead horizon, varying *λ* between 0.01 and 0.1 yields a similar forecasting performance for most models considered. If *λ* is set too large, forecasting accuracy drops. This can be traced back to the fact that a larger penalty leads to an overly sparse model, and this, in turn, decreases the predictive variance too much. The resulting predictive distribution is too narrow, which makes capturing outliers increasingly difficult.

To dig into the sources on why some models work well while others perform poorly when all three variables are jointly considered, we now consider marginal relative LPLs. Considering the density forecasting performance for output, we observe that the model that does well for the joint predictive BF also excels (i.e., the medium‐size VAR with λ=0.01). However, considering the large VARs for output alone reveals that they are also highly competitive and close to the single best performing specification (as long as *λ* is set not too large) and that sparse models often outperform the nonsparse competitor.

Turning to inflation forecasts highlights that they are the main driver of the bad performance of most large‐scale models. Irrespective of the choice of *λ* and *θ*_1_, the small‐scale VAR outperforms each specification and it seems that using SAVS only reduces predictive performance for inflation in large datasets. This result is well known in central bank practice, where these specifications are commonly outperformed by small and medium‐sized models that include only a selected set of endogenous variables (see, e.g., Giannone et al., 2015).

For interest rates, the story found for inflation is reversed. We find that the best models by large margins feature large information sets and are sparse. The main reason behind this strong performance is that the SAVS step sets most coefficients to zero. This yields a predictive distribution for the interest rate, which is strongly centered on zero. During the period of the zero lower bound, the corresponding predictive density will feature a mean/median close to zero with a rather small variance, and this yields large predictive gains in terms of LPLs.

Inspecting multistep‐ahead density forecasting performance yields similar insights to the one‐step‐ahead case. Considering relative joint LPLs indicates that sparse medium‐scale models perform best. The accuracy gains from using SAVS are especially pronounced for this size of the information set. Interestingly, and in contrast to the one‐step‐ahead case, we find that larger values of *λ* (i.e., λ=1 for 1‐year‐ahead and λ=0.5 for 2‐year‐ahead forecasts) translate into the largest gains. Again, we observe that large VARs are generally outperformed by medium‐sized models but, as opposed to one‐quarter‐ahead forecasts, we find that small values of *λ* improve upon the corresponding nonsparse counterpart.

Zooming into variable‐specific performance highlights that large and sparse models yield precise GDP density forecasts that are always better than the nonsparse variant of the model under consideration. These gains are often substantial and sometimes significant at the 10% level. For multistep‐ahead GDP forecasts, we even find that predictive performance increases with model size. This pattern is quite consistent with one exception. The FA‐VAR shows the weakest performance among all models. This indicates that when the researcher wishes to produce multistep‐ahead forecasts, additional information that might be ignored by introducing a factor structure seems to be important.

In terms of inflation, we again see that large models do not perform well, yielding the most inaccurate forecasts across models. Moreover, for multistep‐ahead inflation predictions, we find little evidence that applying SAVS helps to improve forecasts because in most cases the dense model performs better than the sparse variant.

Short‐term interest rates are again most precisely predicted using large models with sparsification. In both cases (1‐ and 2‐year‐ahead), we find that the large VAR with $\theta_{1}=0.075$ and λ=1 performs best. Lower values of *λ* yield highly competitive interest rate forecasts with little differences across the different values of *λ*.

Similarly to the discussion of the point forecasts, we investigate these statements using MCS. Table 5 shows the number of models included in the MCS when we use the negative LPL as a loss function. Starting with an initial set of 33 models we find that, depending on the variable and forecast horizon, a large number of competitors is eliminated. The majority of surviving models is comprised of sparse specifications. While the reduction of models in the case of joint LPLs is moderate to large, we find much smaller MCSs when we focus on variable‐specific forecasting performance. Especially for output and interest rates, we find that the MCS consists of a handful models. In fact, for 2‐year‐ahead predictions, all except for a single model are removed to form the MCS. In both cases, this specification is the large VAR with λ=1 and $\theta_{1}=0.075$. For the remaining cases, the top three of these models can be read of from Table 4 and always includes several sparsified variants. For inflation, the MCS corroborate the findings discussed above. The corresponding set of superior models remains elevated and consequently only a relatively small number of models is eliminated.

**TABLE 5:** Number of models included in the superior models sets (SMSs) for density forecasts according to different shrink‐and‐sparsify specifications

			Joint				Marginal one‐quarter‐ahead				Marginal one‐year‐ahead				Marginal two‐year‐ahead		
			One‐q.‐ahead	One‐y.‐ahead	Two‐y.‐ahead		GDPC1	CPIAUCSL	FEDFUNDS		GDPC1	CPIAUCSL	FEDFUNDS		GDPC1	CPIAUCSL	FEDFUNDS
	MIN		4	1	5		0	6	0		4	6	0		2	6	0
	MIN ‐ λ=0.01		4	2	6		3	5	0		4	6	0		4	6	0
	MIN ‐ λ=0.1		5	3	5		3	6	1		3	5	0		2	6	0
	MIN ‐ λ=0.5		5	2	4		3	6	1		3	6	0		5	6	0
	MIN ‐ λ=1		5	3	5		3	6	1		5	6	1		6	6	1
	SSVS		0	0	2		0	3	0		0	2	0		0	1	0
	Total		23	11	27		12	32	3		19	31	1		19	31	1

*Note*: The SMSs are obtained with the model confidence set (MCS) procedure (Hansen et al., 2011) at a 25% significance level across variables and forecast horizons. The loss function is specified in terms of negative LPLs. For each variable‐horizon combination we use 33 different specifications (including the benchmark). Note that the SSVS prior is only considered for the three smallest information sets.

Before discussing forecasting performance over time, we provide guidance on how to select the penalty parameter *λ* (and thus *ϖ*). As opposed to the simulation exercise, we find that forecast accuracy as measured by relative LPLs differs across specific values of *λ* and forecast horizons. In general, we can recommend setting *λ* to a rather small value (i.e., to 0.01 or 0.1) if interest centers on one‐step‐ahead predictions. This holds for most model sizes. By contrast, if higher‐order forecasts are of interest, we can recommend setting *λ* to a larger value (i.e., 0.5 or 1). With very few exceptions, these are the values that yield the highest LPLs across models. At a first glance, the necessity to discriminate between short and longer run forecasts might point toward a weakness of our approach. However, this consistent pattern can be exploited by setting *λ* as a function of *h* such that it increases with the forecast horizon.

### 5.5 Forecasting performance over time

So far, we computed LPLs over the whole holdout period to provide a measure of average forecast quality. But it could be the case that models have varying performances in different periods. To investigate whether this is indeed the case, we consider density forecast performance over time and across two distinct periods: before the global financial crisis (until 2008:Q1) and thereafter.

**FIGURE 2:**
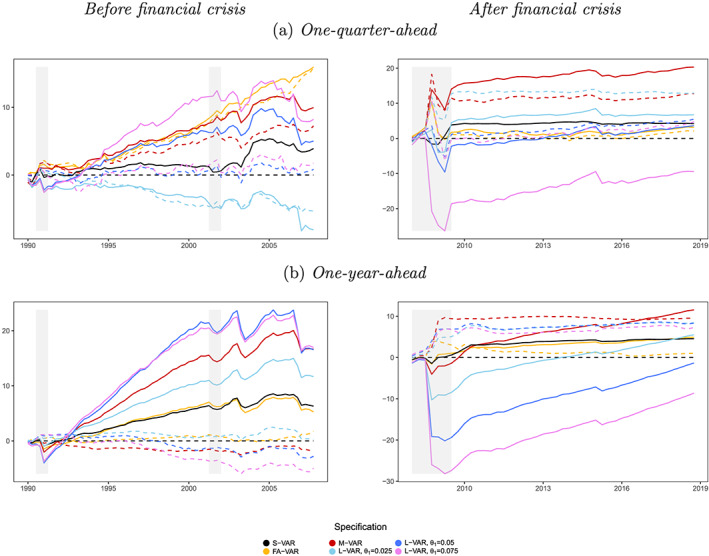
Cumulative joint log predictive likelihoods for 1‐quarter‐ and 1‐year‐ahead predictions benchmarked against the *small‐scale* BVAR without SAVS. Note: Dashed lines indicate classic BVARs while solid lines depict the best performing sparsified version within each information set. Gray shaded areas denote NBER recessions [Colour figure can be viewed at wileyonlinelibrary.com]

Figure 2 shows the evolution of cumulative joint LPLs relative to the benchmark model for 1‐quarter and 1‐year‐ahead predictions over time. The 2‐year‐ahead LPLs look similar and are included in Appendix S3. The figure includes standard BVARs without SAVS (dashed lines) and BVARs post‐processed with an additional SAVS step (solid lines) for all considered information sets. We focus on sparse models with the *λ* that maximizes the LPLs at the end of the hold‐out within each model class (see Table 4). Moreover, for the large‐scale BVAR we depict the evolution of relative LPLs for the different values of *θ*_1_ ∈ {0.025, 0.05, 0.075}.

Considering the period before the financial crisis clearly shows that using SAVS often yields more precise forecast distributions. This finding is especially pronounced for higher order forecasts, where we see strong and sustained gains over the period up to the financial crisis. The performance of the sparsified large models with $\theta_{1}=\{0.075,0.05\}$ and the medium‐scale BVAR with SAVS appear to be the best specifications during this time span.

When we focus attention on the global financial crisis, we observe a pronounced decline in model evidence for the large model with SAVS (right panels in Figure 2). During the crisis, evidence in favor of sparsification also slightly decreases for the medium‐scale model. This can be seen by comparing the solid and dashed red lines in Figure 2. While the nonsparse, medium‐sized BVAR is outperformed in the run‐up to the crisis, model evidence marginally supports the nonsparse variant during the recession. The main reason why predictive accuracy of sparsified models deteriorates in turbulent periods is that the forecast error variance becomes too small and large shocks become increasingly unlikely under the predictive distribution. In such situations, dense models often feature a larger variance because of many small but nonzero $v_{ij}^{\prime }$s in Equation (10). This makes capturing outliers (or rapid shifts) easier and thus improves LPLs.

After the financial crisis, we see that applying SAVS helps for some models (especially the medium‐scale VAR). However, predictive evidence in favor of SAVS declines with the forecast horizon. This pattern is the opposite of the one observed before the financial crisis. But note that this is mostly driven by the dismal performance during the recession. For 1‐year‐ahead forecasts, we observe that the slopes of most LPL curves relative to sparse models are steep, indicating a period‐by‐period outperformance vis‐á‐vis the benchmark model.

### 5.6 Assessing model calibration using probability integral transforms

In this section, we investigate how sparsification impacts the calibration of predictive densities. Therefore, we follow Giordani and Villani (2010) and Clark (2011) in analyzing normalized forecast errors that are obtained by applying the probability integral transform (PIT). 11If a given model is well calibrated, these normalized forecast errors should be standard normally distributed. Deviations from the standard normal distribution provide information along which dimension the model might be misspecified.

**FIGURE 3:**
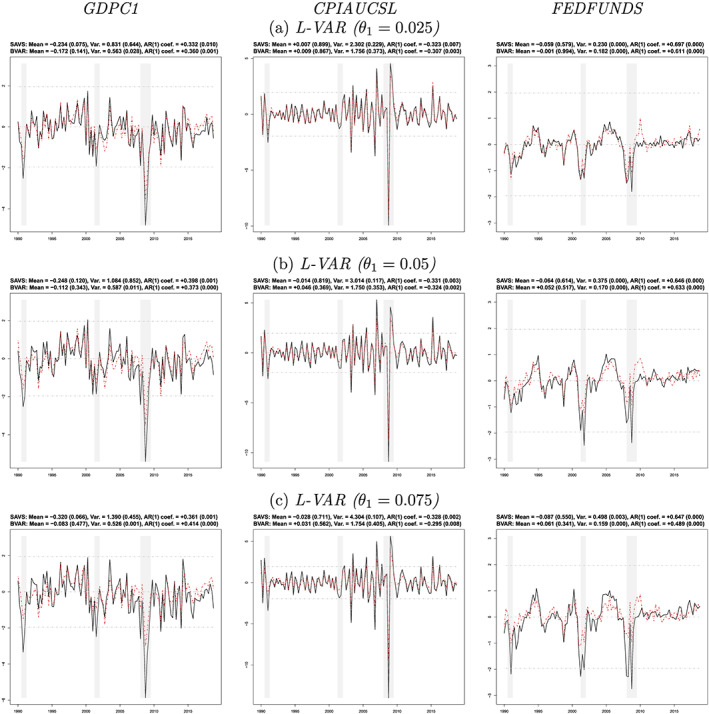
Normalized 1‐year‐ahead forecast errors of large‐scale models. Note: The black solid lines represent the sparsified versions (SAVS) with λ=1 while the red dash‐dotted lines depict classic BVARs. The gray dash‐dotted horizontal lines indicate the 95*%* interval of the standard normal distribution and the gray shaded areas denote NBER recessions. Moreover, the legends show the corresponding test statistics of normalized errors. We follow Clark (2011) and show the mean, the variance (Var.), and the autoregressive coefficient (AR(1) coef.) of normalized errors. In parenthesis we depict the corresponding *p*‐values. The null‐hypotheses, a zero mean, a variance of one, and no autocorrelated errors, are tested separately [Colour figure can be viewed at wileyonlinelibrary.com] [Correction added on 19 February 2021, after first online publication: Figure 3 has been updated in this version.]

We focus on 1‐year‐ahead predictive densities for brevity. Moreover, in the main text, we focus on the large‐scale models. 12In Figure 3, we compare the 1‐year‐ahead normalized forecast errors for the large sparse VAR (with λ=1) to the nonsparsified BVAR model for the three target variables. Because this purely visual analysis might be misleading, we add a legend to each panel that provides information on whether departures from standard normality are statistically significant. The null hypothesis is that the normalized forecast errors are zero mean, feature a variance of one and no serial correlation (Berkowitz, 2001). 13

Before focusing on sparsified models, we analyze the nonsparsified counterparts. For the standard BVAR forecasts, the mean is very often not significantly different from zero. With regards to the variance we find that normalized forecast errors display variances well below unity for both GDP and the interest rate, while displaying a particularly high variance for inflation forecasts. A variance of forecast errors below one indicates that for many periods in the hold‐out, the predictive distribution is too spread out. As discussed above, this may be at least in part attributed to the imprecisely estimated small parameters. By contrast, a variance above one would indicate that the predictive density tends to be too tight. Moreover, we observe a high autocorrelation of forecast errors for the interest rate. This feature is also commonly found in the literature (see, e.g., Clark, 2011).

If we apply the additional SAVS step, the properties of the normalized forecast errors often improve. In the case of GDP, sparsifying the BVAR typically increases the variances at a small cost of a slightly more negative mean (which is not significantly different from zero in almost all instances). For inflation, and especially during the financial crisis, sparsification does not seem to help. The nonsparsified BVAR estimates tend to produce already too tight predictive distributions. These become even tighter after applying sparsification. For the interest rate, BVAR models produce normalized forecast errors with variances well below unity. Using large VARs coupled with a relative loose prior and adding SAVS improves this. More precisely, for the sparsified large VAR with $\theta_{1}=0.075$, we find that the variance increases from 0.16 to approximately 0.5. Although this is still below the unit variance we would expect under a well calibrated model, we consider this a substantial improvement at the costs of slightly higher autocorrelation in the errors.

In general, the PITs suggest that using SAVS has the potential to improve model calibration for some models and most variables. A recommendation for practitioners might be that if the variance of normalized forecast errors of a BVAR is well below one, SAVS can substantially improve forecast density calibration (see, e.g., the normalized errors for output and interest rate forecasts). Conversely, if the predictive variance of a BVAR is already too tight, characterized by normalized forecast errors that feature a variance above one, SAVS tends to hurt predictive accuracy (see the case of inflation).

## 6 CONCLUSIONS

This paper proposes methods to shrink‐and‐sparsify VAR models with conjugate priors. The main feature of our SAVS approach is that we postprocess each draw from the joint posterior by solving an optimization problem to search for a sparse coefficient vector. Without breaking the conjugacy of the model, this approach allows for different predictors across the equations in the VAR. And, instead of pushing coefficients close to zero, our approach introduces exact zeros, removing the lower bound on accuracy one can achieve under a popular shrinkage prior in the Minnesota tradition. Because the error covariance matrix in large VARs also features a large number of coefficients, we adapt techniques from the literature on graphical models to obtain a sparse estimate of the variance–covariance matrix of the system.

Using synthetic data, we show that combining shrinkage and sparsity pays off in large models with DGPs being sparse. These improvements in additional estimation accuracy come with little additional computational costs. In our real‐data application, we investigate the forecasting properties of our sparse VARs. Using U.S. data, we show that although our methods do not substantially improve point forecasts of output, inflation, and interest rates, density forecasts arising from the sparse models are often much better than those obtained from the shrinkage‐alone models.

### OPEN RESEARCH BADGES

This article has earned an Open Data Badge for making publicly available the digitally‐shareable data necessary to reproduce the reported results. The data is available at [http://qed.econ.queensu.ca/jae/datasets/hauzenberger001/].

## Supporting information



The JAE Data Archive directory is available at http://qed.econ.queensu.ca/jae/datasets/hauzenberger001/

Supporting info item

## Footnotes

1 For recent solutions that allow for estimating large‐scale VARs under nonconjugate priors, see Carriero, Clark, and Marcellino (2019).

2 For higher‐order forecasts or other quantities such as impulse responses, Monte Carlo simulation is necessary.

3 Strictly speaking, this is the solution obtained after the first iteration of the optimization algorithm, which, conditional on initializing the algorithm at the posterior mean, already indicates convergence at this stage.

4 In the empirical application, we specify the penalty on the intercept term equal to zero.

5 Note that, if ${\hat{\omega }}_{ij}^{*}$ with *i* ≠ *j*, the (*i*, *j*)th element of ${\hat{\Omega }}^{*}$, is set to zero, then *y*_*it*_ and *y*_*jt*_ exhibit no contemporaneous relationship.

6 More precise results based on averages of the loss functions of the optimization routine are available in Figure S1.

7 As alternative metric, we also compute continuous rank probability scores (CRPS). Compared with the LPLs, the CRPS yield qualitatively similar insights. For the sake of brevity, we focus on LPLs in the paper. The results for CRPS can be found in Table S4.

8 In Appendix S1, we show a detailed list of the variables included along the transformation codes.

9 To circumvent this issue, Bańbura et al. (2010) and Koop (2013), for example, define a training sample which serves the purpose to calibrate *θ*_1_ by minimizing the distance of the mean square error (MSE) of a large‐scale model and a three‐variable VAR estimated with OLS. Intuitively, this strategy implies that large dimensional models are shrunk to a larger degree than smaller‐scale models (De Mol, Giannone, & Reichlin, 2008).

10 Note that the test statistics must be interpreted with caution, because we use a recursive forecast design.

11 For more details on how the normalized forecast errors are computed, see Clark (2011).

12 The normalized forecast errors for the other forecast horizons and model sizes are available in the Online Appendix.

13 Following Clark (2011), the *p* value of a zero mean is computed with a Newey–West variance with five lags, the *p* value of a unit variance is obtained by regressing the squared normalized forecast errors on an intercept and using a Newey–West variance with three lags, and the *p* value of no autocorrelation is computed with an AR(1) model, featuring an unconditional mean and heteroskedasticity‐robust standard errors.
