# Supplementary material for: Combining shrinkage and sparsity in conjugate vector autoregressive models
Source: J Appl Econ (Chichester Engl). 2021 Jan 13;36(3):304–27. doi: 10.1002/jae.2807 (PMC8048898; doi:10.1002/jae.2807)
Supplement: Supplementary file 1 — Supporting info item [file JAE-36-304-s001.pdf]

Online Appendix to: "Combining Shrinkage and Sparsity in Conjugate Vector Autoregressive Models"

NIKO HAUZENBERGER[1], FLORIAN HUBER*[1], and LUCA ONORANTE[2]

[1] *University of Salzburg*
[2] *Joint Research Centre, European Commission*

October 21, 2020

*Corresponding author: Florian Huber. Department of Economics and Salzburg Centre of European Union Studies, University of Salzburg. Address: Mönchsberg 2a, 5020 Salzburg, Austria. Email: florian.huber@sbg.ac.at. The first two authors gratefully acknowledge financial support by the Austrian Science Fund (FWF): ZK 35 and by funds of the Oesterreichische Nationalbank (Austrian Central Bank, Anniversary Fund, project number: 18127). We would like to thank Michael McCracken, three anonymous reviewers, Michael Pfarrhofer and Gary Koop for helpful comments and suggestions that improved the quality of the paper substantially.

A Data description

Here, we provide detailed information on the transformation applied for each variable, as we transform the data to stationarity, according to the suggestions of McCracken and Ng (2016). With stationary data the prior is centered on zero, assuming a white noise process for each variable a priori. Moreover, we standardise the data by demeaning each variable and dividing through the standard deviation. Due to the scale-variance of PCs the data is also standardised before extracting the factors.

Table A1: Data description

FRED.Mnemonic	Description	Trans I(0)	S	M	L
**Slow**					
GDPC1	Real Gross Domestic Product	5	x	x	x
PCECC96	Real Personal Consumption Expenditures	5		x	x
PCDGx	Real personal consumption expenditures: Durable goods	5			x
PCESVx	Real Personal Consumption Expenditures: Services	5			x
PCNDx	Real Personal Consumption Expenditures: Nondurable Goods	5			x
GPDIC1	Real Gross Private Domestic Investment	5			x
FPIx	Real private fixed investment	5		x	x
Y033RC1Q027SBEAx	Real Gross Private Domestic Investment: Fixed Investment: Nonresidential Equipment	5			x
PNFIx	Real private fixed investment: Nonresidential	5			x
PRFIx	Real private fixed investment: Residential	5			x
A014RE1Q156NBEA	Shares of gross domestic product: Gross private domestic investment: Change in private inventories	1			x
GCEC1	Real Government Consumption Expenditures and Gross Investment	5		x	x
A823RL1Q225SBEA	Real Government Consumption Expenditures and Gross Investment: Federal	1			x
FGRECPTx	Real Federal Government Current Receipts	5			x
SLCEx	Real government state and local consumption expenditures	5			x
EXPGSC1	Real Exports of Goods and Services	5			x
IMPGSC1	Real Imports of Goods and Services	5			x
DPIC96	Real Disposable Personal Income	5			x
OUTNFB	Nonfarm Business Sector: Real Output	5			x
OUTBS	Business Sector: Real Output	5			x
INDPRO	IP:Total index Industrial Production Index (Index 2012=100)	5		x	x
IPFINAL	IP:Final products Industrial Production: Final Products (Market Group) (Index 2012=100)	5			x
IPCONGD	IP:Consumer goods Industrial Production: Consumer Goods (Index 2012=100)	5			x
IPMAT	Materials (Index 2012=100)	5			x
IPDMAT	Durable Materials (Index 2012=100)	5			x
IPNMAT	Nondurable Materials (Index 2012=100)	5			x
IPDCONGD	Durable Consumer Goods (Index 2012=100)	5			x
IPB51110SQ	Durable Goods: Automotive products (Index 2012=100)	5			x
IPNCONGD	Nondurable Consumer Goods (Index 2012=100)	5			x
IPBUSEQ	Business Equipment (Index 2012=100)	5			x
IPB51220SQ	Consumer energy products (Index 2012=100)	5			x
CUMFNS	Capacity Utilization: Manufacturing (SIC) (Percent of Capacity)	1			x
IPMANSICS	Industrial Production: Manufacturing (SIC) (Index 2012=100)	5			x
IPB51222S	Industrial Production: Residential Utilities (Index 2012=100)	5			x
IPFUELS	Industrial Production: Fuels (Index 2012=100)	5			x
PAYEMS	Emp:Nonfarm All Employees: Total nonfarm (Thousands of Persons)	5			x
USPRIV	All Employees: Total Private Industries (Thousands of Persons)	5			x
MANEMP	All Employees: Manufacturing (Thousands of Persons)	5			x
SRVPRD	All Employees: Service-Providing Industries (Thousands of Persons)	5			x
USGOOD	All Employees: Goods-Producing Industries (Thousands of Persons)	5			x
DMANEMP	All Employees: Durable goods (Thousands of Persons)	5			x
NDMANEMP	All Employees: Nondurable goods (Thousands of Persons)	5			x
USCONS	All Employees: Construction (Thousands of Persons)	5			x
USEHS	All Employees: Education & Health Services (Thousands of Persons)	5			x
USFIRE	All Employees: Financial Activities (Thousands of Persons)	5			x
USINFO	All Employees: Information Services (Thousands of Persons)	5			x
USPBS	All Employees: Professional & Business Services (Thousands of Persons)	5			x
USLAH	All Employees: Leisure & Hospitality (Thousands of Persons)	5			x
USSERV	All Employees: Other Services (Thousands of Persons)	5			x
USMINE	All Employees: Mining and logging (Thousands of Persons)	5			x
USTPU	All Employees: Trade, Transportation & Utilities (Thousands of Persons)	5			x
USGOVT	All Employees: Government (Thousands of Persons)	5			x
USTRADE	All Employees: Retail Trade (Thousands of Persons)	5			x
USWTRADE	All Employees: Wholesale Trade (Thousands of Persons)	5			x
CES9091000001	All Employees: Government: Federal (Thousands of Persons)	5			x
CES9092000001	All Employees: Government: State Government (Thousands of Persons)	5			x
CES9093000001	All Employees: Government: Local Government (Thousands of Persons)	5			x
CE16OV	Civilian Employment (Thousands of Persons)	5		x	x
CIVPART	Civilian Labor Force Participation Rate (Percent)	2			x
UNRATE	Civilian Unemployment Rate (Percent)	2		x	x
UNRATESTx	Unemployment Rate less than 27 weeks (Percent)	2			x
UNRATELTx	Unemployment Rate for more than 27 weeks (Percent)	2			x
LNS14000012	Unemployment Rate - 16 to 19 years (Percent)	2			x
LNS14000025	Unemployment Rate - 20 years and over, Men (Percent)	2			x
LNS14000026	Unemployment Rate - 20 years and over, Women (Percent)	2			x
UEMPLT5	Number of Civilians Unemployed - Less Than 5 Weeks (Thousands of Persons)	5			x
UEMP5TO14	Number of Civilians Unemployed for 5 to 14 Weeks (Thousands of Persons)	5			x
UEMP15T26	Number of Civilians Unemployed for 15 to 26 Weeks (Thousands of Persons)	5			x
UEMP27OV	Number of Civilians Unemployed for 27 Weeks and Over (Thousands of Persons)	5			x
AWHMAN	Average Weekly Hours of Production and Nonsupervisory Employees: Manufacturing (Hours)	1			x
AWOTMAN	Average Weekly Overtime Hours of Production and Nonsupervisory Employees: Manufacturing (Hours)	2			x
HWIx	Help-Wanted Index	1			x
CES0600000007	Average Weekly Hours of Production and Nonsupervisory Employees: Goods-Producing	2		x	x
CLAIMSx	Initial Claims	5			x
HOUST	Housing Starts: Total: New Privately Owned Housing Units Started	5		x	x

Table A2: Data description (cont.)

FRED.Mnemonic	Description	Trans I(0)	S	M	L
**Slow**					
HOUST5F	Privately Owned Housing Starts: 5-Unit Structures or More	5			x
PERMIT	New Private Housing Units Authorized by Building Permits	5		x	x
HOUSTMW	Housing Starts in Midwest Census Region (Thousands of Units)	5			x
HOUSTNE	Housing Starts in Northeast Census Region (Thousands of Units)	5			x
HOUSTS	Housing Starts in South Census Region (Thousands of Units)	5			x
HOUSTW	Housing Starts in West Census Region (Thousands of Units)	5			x
RSAFSx	Real Retail and Food Services Sales (Millions of Chained 2012 Dollars)	5			x
AMDMNOx	Real Manufacturers' New Orders: Durable Goods (Millions of 2012 Dollars)	5			x
AMDMUOx	Real Value of Manufacturers' Unfilled Orders for Durable Goods Industries	5			x
BUSINVx	Total Business Inventories (Millions of Dollars)	5			x
ISRATIOx	Total Business: Inventories to Sales Ratio	2			x
PCECTPI	Personal Consumption Expenditures: Chain-type Price Index	6		x	x
PCEPILFE	Personal Consumption Expenditures Excluding Food and Energy	6			x
GDPCTPI	Gross Domestic Product: Chain-type Price Index	5	x	x	x
GPDICTPI	Gross Private Domestic Investment: Chain-type Price Index	6			x
IPDBS	Business Sector: Implicit Price Deflator (Index 2012=100)	6			x
DGDSRG3Q086SBEA	Personal consumption expenditures: Goods	6			x
DDURRG3Q086SBEA	Personal consumption expenditures: Durable goods	6			x
DSERRG3Q086SBEA	Personal consumption expenditures: Services	6			x
DNDGRG3Q086SBEA	Personal consumption expenditures: Nondurable goods	6			x
DHCERG3Q086SBEA	Personal consumption expenditures: Services: Household consumption expenditures	6			x
DMOTRG3Q086SBEA	Personal consumption expenditures: Durable goods: Motor vehicles and parts	6			x
DFDHRG3Q086SBEA	Personal consumption expenditures: Durable goods: Furnishings and durable household equipment	6			x
DREQRG3Q086SBEA	Personal consumption expenditures: Durable goods: Recreational goods and vehicles	6			x
DODGRG3Q086SBEA	Personal consumption expenditures: Durable goods: Other durable goods	6			x
DFXARG3Q086SBEA	Personal consumption expenditures: Nondurable goods: Food and beverages purchased for off-premises consumption	6			x
DCLORG3Q086SBEA	Personal consumption expenditures: Nondurable goods: Clothing and footwear	6			x
DGOERG3Q086SBEA	Personal consumption expenditures: Nondurable goods: Gasoline and other energy goods	6			x
DONGRG3Q086SBEA	Personal consumption expenditures: Nondurable goods: Other nondurable goods	6			x
DHUTRG3Q086SBEA	Personal consumption expenditures: Services: Housing and utilities	6			x
DHLCRG3Q086SBEA	Personal consumption expenditures: Services: Health care	6			x
DTRSRG3Q086SBEA	Personal consumption expenditures: Transportation services	6			x
DRCARG3Q086SBEA	Personal consumption expenditures: Recreation services	6			x
DFSARG3Q086SBEA	Personal consumption expenditures: Services: Food services and accomodations	6			x
DIFSRG3Q086SBEA	Personal consumption expenditures: Financial services and insurance	6			x
DOTSRG3Q086SBEA	Personal consumption expenditures: Other services	6			x
CPIAUCSL	Consumer Price Index for All Urban Consumers: All Items	6		x	x
CPILFESL	Consumer Price Index for All Urban Consumers: All Items Less Food & Energy	6			x
WPSFD49207	Producer Price Index by Commodity for Finished Goods	6			x
PPIACO	Producer Price Index for All Commodities	6			x
WPSFD49502	Producer Price Index by Commodity for Finished Consumer Goods	6			x
WPSFD4111	Producer Price Index by Commodity for Finished Consumer Foods	6			x
PPIIDC	Producer Price Index by Commodity Industrial Commodities	6			x
WPSID61	Producer Price Index by Commodity Intermediate Materials: Supplies & Components	6			x
WPU0561	Producer Price Index by Commodity for Fuels and Related Products and Power	5			x
OILPRICEx	Real Crude Oil Prices: West Texas Intermediate (WTI) - Cushing, Oklahoma	5			x
WPSID62	Producer Price Index: Crude Materials for Further Processing	6			x
PPICMM	Producer Price Index: Commodities: Metals and metal products: Primary nonferrous metals	6			x
CPIAPPSL	Consumer Price Index for All Urban Consumers: Apparel	6			x
CPITRNSL	Consumer Price Index for All Urban Consumers: Transportation	6			x
CPIMEDSL	Consumer Price Index for All Urban Consumers: Medical Care	6			x
CUSR0000SAC	Consumer Price Index for All Urban Consumers: Commodities	6			x
CES2000000008x	Real Average Hourly Earnings of Production and Nonsupervisory Employees: Construction	5			x
CES3000000008x	Real Average Hourly Earnings of Production and Nonsupervisory Employees: Manufacturing	5			x
COMPRNFB	Nonfarm Business Sector: Real Compensation Per Hour (Index 2012=100)	5			x
CES0600000008	Average Hourly Earnings of Production and Nonsupervisory Employees:	6		x	x
**Policy rate**					
FEDFUNDS	Effective Federal Funds Rate (Percent)	2	x	x	x
**Fast**					
TB3MS	3-Month Treasury Bill: Secondary Market Rate (Percent)	2			x
TB6MS	6-Month Treasury Bill: Secondary Market Rate (Percent)	2			x
GS1	1-Year Treasury Constant Maturity Rate (Percent)	2		x	x
GS10	10-Year Treasury Constant Maturity Rate (Percent)	2		x	x
AAA	Moody's Seasoned Aaa Corporate Bond Yield (Percent)	2			x
BAA	Moody's Seasoned Baa Corporate Bond Yield (Percent)	2			x
BAA10YM	Moody's Seasoned Baa Corporate Bond Yield Relative to Yield on 10-Year Treasury	1			x
TB6M3Mx	6-Month Treasury Bill Minus 3-Month Treasury Bill, secondary market (Percent)	1			x
GS1TB3Mx	1-Year Treasury Constant Maturity Minus 3-Month Treasury Bill, secondary market	1			x
GS10TB3Mx	10-Year Treasury Constant Maturity Minus 3-Month Treasury Bill, secondary market	1			x
CPF3MTB3Mx	3-Month Commercial Paper Minus 3-Month Treasury Bill, secondary market	1			x
GS5	5-Year Treasury Constant Maturity Rate	2			x
TB3SMFFM	3-Month Treasury Constant Maturity Minus Federal Funds Rate	1			x
T5YFFM	5-Year Treasury Constant Maturity Minus Federal Funds Rate	1			x
AAAFFM	Moody's Seasoned Aaa Corporate Bond Minus Federal Funds Rate	1			x
BUSLOANSx	Real Commercial and Industrial Loans, All Commercial Banks	5			x
CONSUMERx	Real Consumer Loans at All Commercial Banks	5			x
NONREVSLx	Total Real Nonrevolving Credit Owned and Securitized, Outstanding	5			x
REALLNx	Real Real Estate Loans, All Commercial Banks	5			x
TOTALSLx	Total Consumer Credit Outstanding	5			x
TOTRESNS	Total Reserves of Depository Institutions	6		x	x
NONBORRES	Reserves Of Depository Institutions, Nonborrowed	7		x	x
DTCOLNVHFNM	Consumer Motor Vehicle Loans Outstanding Owned by Finance Companies	6			x
DTCTHFNM	Total Consumer Loans and Leases Outstanding Owned and Securitized by Finance Companies	6			x
INVEST	Securities in Bank Credit at All Commercial Banks	6			x
TABSHNOx	Real Total Assets of Households and Nonprofit Organizations	5			x
EXSZUSx	Switzerland / U.S. Foreign Exchange Rate	5			x
EXJPUSx	Japan / U.S. Foreign Exchange Rate	5			x
EXUSUKx	U.S. / U.K. Foreign Exchange Rate	5			x
EXCAUSx	Canada / U.S. Foreign Exchange Rate	5			x
S.P.500	S&P's Common Stock Price Index: Composite	5		x	x
S.P..indust	S&P's Common Stock Price Index: Industrials	5			x
S.P.div.yield	S&P's Composite Common Stock: Dividend Yield	2			x

B Additional Simulation-based Results

B.1 Runtime comparison

Table B1 shows relative computation times of the different model sizes considered in the simulation exercise. The table highlights that adding the additional SAVS step increases computation times only marginally (from around 1% to 20%). It is worth stressing that the absolute estimation times of the large conjugate BVAR model are small and, even more important, that the additional SAVS step becomes computationally less costly with increasing m in relative terms. This is clearly not the case for the SSVS-VAR. Especially in larger dimensions, estimating the SSVS model becomes quickly infeasible. For instance, in the large DGP ($m = 30$) and with $T = 240$, obtaining $10,000$ draws from the posterior for the non-conjugate VAR takes around 63 minutes.

Table B1: Runtime comparison of simulation study across m and T with $10,000$ draws from the posterior distribution.

**DGP**		**in mins.**	**relative to BVAR**		
m	T	BVAR	SAVS	CDA	SSVS
**S**	80	0.814	1.116	1.209	1.053
	240	0.856	1.141	1.245	1.245
**M**	80	2.011	1.053	1.953	3.168
	240	2.097	1.082	2.982	3.649
**L**	80	12.902	1.008	4.867	3.693
	240	13.541	1.013	4.192	4.631

Notes: Runtimes are based on two IntelE5-2650v3 2,3 GHz cores and 256GB RAM.

B.2 Comparing the loss of SAVS to the coordinate descent algorithm

The coordinate descent algorithm (CDA) serves to illustrate whether using more iterations yields similar insights compared to an algorithm that stops after the first iteration. To provide additional evidence, Figure B1 shows the loss averaged across draws for each iteration of the CDA. In panel (a) we consider a quite dense DGP while in panel (b) we consider a very sparse one. To compute the loss for each draw, we simply plug-in the sparsified draw after each iteration in the loss/objective functions of both $\boldsymbol{a}$ and $\boldsymbol{\Sigma}^{-1}$. Similar to Ray and Bhattacharya (2018), we find evidence that the coordinate descent algorithm already converges after the first iteration when applying it to draws.

Figure B1: Loss averaged across draws for each iteration of a coordinate descent algorithm (CDA).

a) $m = 30$, $T = 240$, and degree of sparsity 10%   b) $m = 30$, $T = 240$, and degree of sparsity 90%

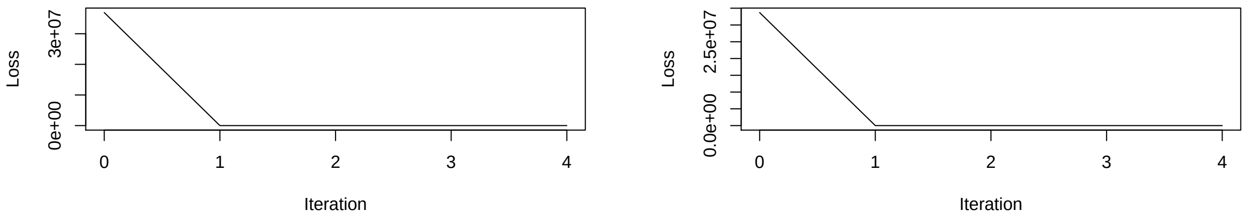

Notes: SAVS implies stopping after the first iteration.

C Additional Forecasting Results

Figure C1: Cumulative joint log predictive likelihoods for two-year-ahead predictions benchmarked against the *small-scale* BVAR without SAVS.

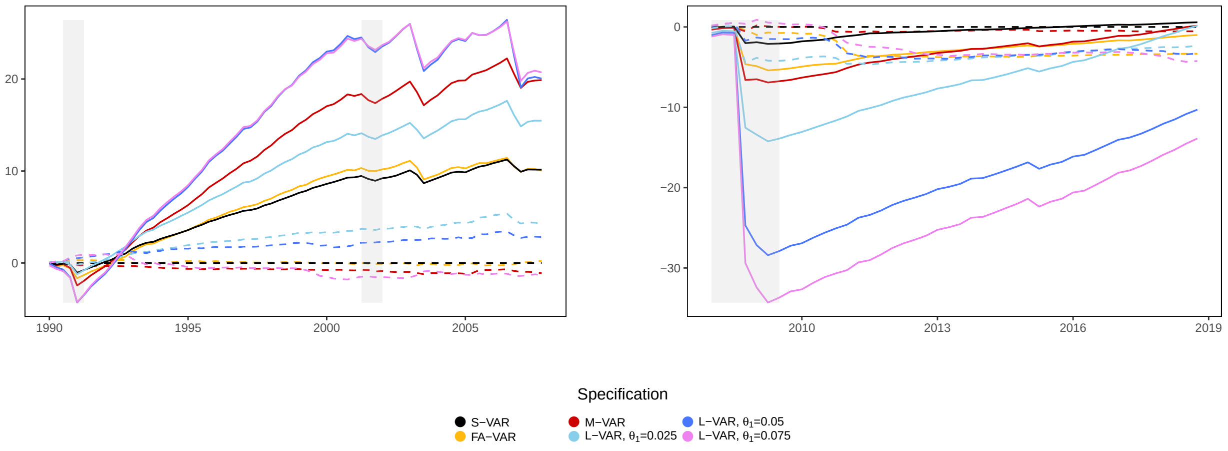

Notes: Dashed lines indicate classic BVARs while solid lines depict the best performing sparsified version within each information set. Gray shaded areas denote NBER recessions.

Figure C2: Normalized one-year-ahead forecast errors of small and moderate sized models.

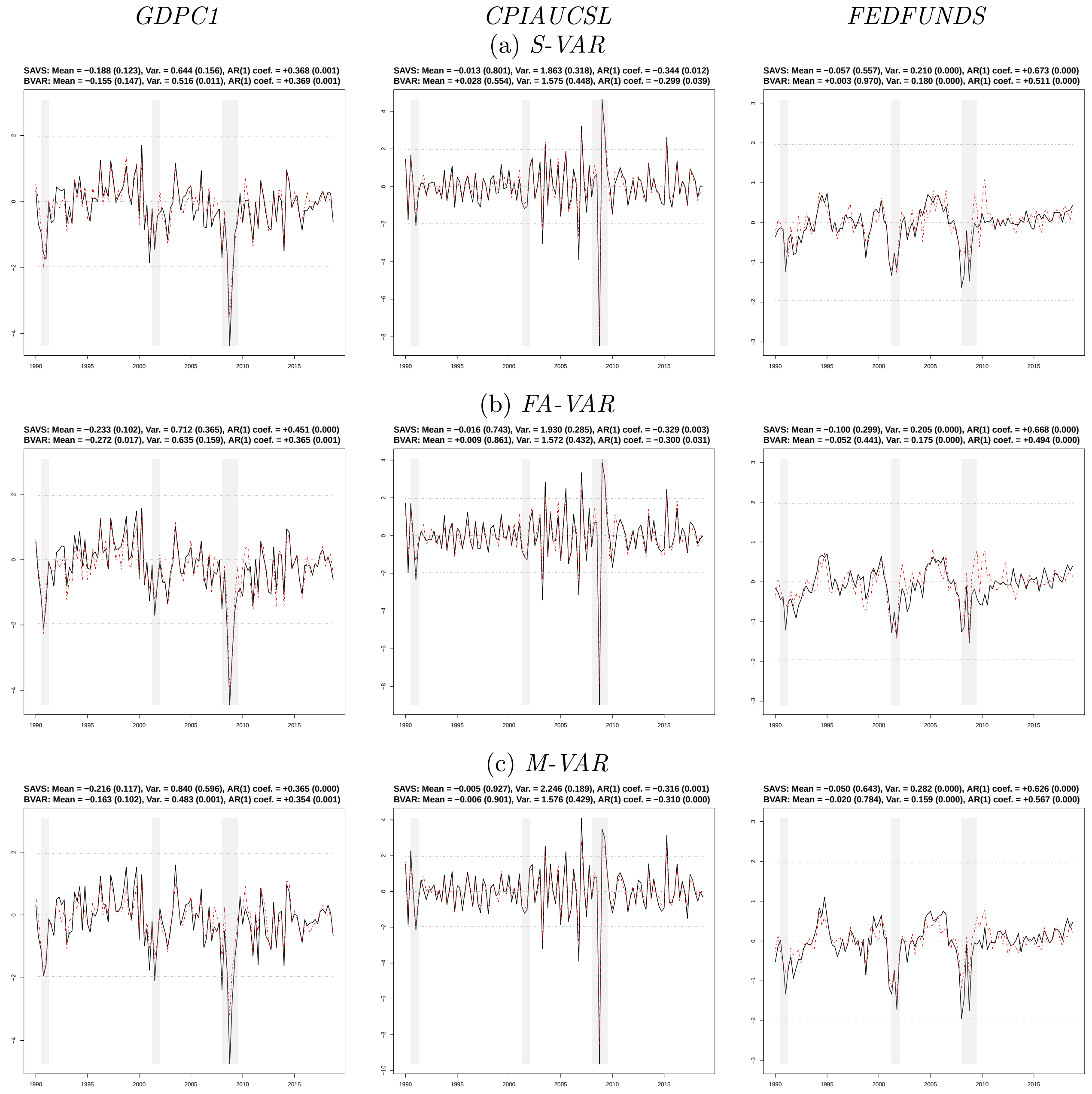

Notes: The black solid lines represent the sparsified versions (SAVS) with $\lambda = 1$ while the red dash-dotted lines depict classic BVARs. The gray dash-dotted horizontal lines indicate the 95% interval of the standard normal distribution and the gray shaded areas denote NBER recessions. Moreover, the legends show the corresponding test statistics of normalized errors. Here we follow (Clark, 2011) and show the mean, the variance (Var.), and the autoregressive coefficient (AR(1) coef.) of normalized errors. In parenthesis we depict the corresponding p-values. The null-hypotheses, a zero mean, a variance of one, and no autocorrelated errors, are tested separately.

Table C1: CRPS ratios relative to the *small-scale* BVAR with a Minnesota prior.

Specification	Average			Marginal One-quarter-ahead			Marginal One-year-ahead			Marginal Two-year-ahead		
	One-q.-ahead	One-y.-ahead	Two-y.-ahead	GDPC1	CPIAUCSL	FEDFUNDS	GDPC1	CPIAUCSL	FEDFUNDS	GDPC1	CPIAUCSL	FEDFUNDS
**S-VAR**												
MIN - $\lambda = 0.01$	0.984	1.002	0.989	0.997	**0.980 (1)**	0.975	1.011	0.986	1.023	0.979	0.989	1.002
MIN - $\lambda = 0.1$	0.971 (2)	0.996	0.981***	0.986	0.990 (3)	0.917*	1.008	0.999	0.971	0.959**	0.993	0.984
MIN - $\lambda = 0.5$	**0.967 (1)**	0.982	0.986	0.992	1.000	0.876**	0.979	0.987	0.973	0.982	0.983	1.000
MIN - $\lambda = 1$	0.976	0.976	0.971***	0.997	1.021	0.867**	0.976	0.994	0.940	0.963*	0.975*	0.974
SSVS	1.003	1.025*	1.016**	1.018	0.984 (2)	1.017	1.044	1.013	1.026	1.034**	0.984	1.061***
**FA-VAR**												
MIN	1.013	1.031**	1.008	0.956	1.052*	1.018	1.069**	1.025	0.994	1.070*	0.980	0.983
MIN - $\lambda = 0.01$	0.997	1.015	0.999	0.939	1.038	0.998	1.036	1.018	0.982	0.990	0.996	1.016
MIN - $\lambda = 0.1$	0.996	1.012	0.978**	0.939	1.062	0.951	1.068*	0.990	0.985	0.973	0.972* (3)	0.997
MIN - $\lambda = 0.5$	1.010	1.010	0.982**	0.943	1.095	0.946	1.076	**0.971 (1)**	1.002	0.964*	0.992	0.987
MIN - $\lambda = 1$	1.022	0.997	0.983	0.937	1.118	0.959	1.009	0.992	0.993	0.952**	0.994	1.001
SSVS	1.284***	1.275***	1.263***	0.922	1.583***	1.214**	1.112***	1.454***	1.124*	1.072**	1.424***	1.184***
**M-VAR**												
MIN	1.020	0.998	1.011	0.881** (3)	1.136*	0.992	1.020	0.982	1.003	1.027	0.993	1.027
MIN - $\lambda = 0.01$	1.001	0.968	0.981	0.868*** (2)	1.145*	0.911	0.972	0.977 (3)	0.947	0.976	0.988	0.972
MIN - $\lambda = 0.1$	0.974	0.967	0.958***	**0.859*** (1)**	1.138*	0.830***	0.965	0.986	0.934	0.950	0.979	0.925***
MIN - $\lambda = 0.5$	0.972 (3)	0.954	0.933***	0.928**	1.091	0.814***	0.958	0.983	0.889*	0.931***	**0.956*** (1)**	0.887***
MIN - $\lambda = 1$	0.988	0.947*	0.951***	0.972	1.091	0.823***	0.951	0.984	0.869**	0.942**	0.984	0.893***
SSVS	1.339***	1.315***	1.356***	0.980	1.653***	1.235***	1.132***	1.482***	1.213**	1.178*	1.474***	1.349**
**L-VAR** ($\theta_1 = 0.025$)												
MIN	1.001	0.986	0.983	0.895**	1.105	0.950	1.004	0.981	0.972	0.999	0.974**	0.980
MIN - $\lambda = 0.01$	1.020	0.955	0.955***	0.969	1.134*	0.882**	0.966	0.974 (2)	0.903*	0.945**	0.979*	0.916***
MIN - $\lambda = 0.1$	1.034	0.958	0.955***	0.993	1.125	0.922	0.973	0.983	0.886**	0.945*	0.979	0.920**
MIN - $\lambda = 0.5$	1.025	0.957	0.955***	1.005	1.103	0.910	0.959	0.988	0.894*	0.925***	0.988	0.924***
MIN - $\lambda = 1$	1.034	0.963	0.950***	1.006	1.117	0.921	0.969	0.994	0.891**	0.935***	0.973*	0.922**
**L-VAR** ($\theta_1 = 0.05$)												
MIN	1.003	0.994	0.989	0.922	1.081	0.966	1.026	0.980	0.982	1.009	0.984	0.972
MIN - $\lambda = 0.01$	0.985	0.948*	0.938***	0.903***	1.128	0.834***	0.955	0.984	0.865**	0.936***	0.969* (2)	0.874***
MIN - $\lambda = 0.1$	1.015	0.940**	0.933***	0.970	1.140*	0.849**	0.949	0.989	0.828***	0.926**	0.985	0.830***
MIN - $\lambda = 0.5$	1.002	0.940**	0.932***	0.980	1.108	0.840**	0.947	0.995	0.820***	0.924**	0.980	0.842***
MIN - $\lambda = 1$	1.005	0.939**	0.932***	0.980	1.115	0.840**	0.944 (2)	0.994	0.821***	0.925**	0.978*	0.844***
**L-VAR** ($\theta_1 = 0.075$)												
MIN	1.017	1.001	1.024	0.943	1.085	0.989	1.020	0.988	1.000	1.073*	0.993	1.022
MIN - $\lambda = 0.01$	0.995	0.935***	0.934***	0.905**	1.146*	0.841***	0.944** (3)	0.980	0.833***	0.923*** (3)	0.980	0.854***
MIN - $\lambda = 0.1$	0.993	0.930** (2)	0.928*** (3)	0.965	1.133*	0.780*** (2)	0.948	0.992	0.778*** (3)	0.915** (2)	0.993	0.809*** (3)
MIN - $\lambda = 0.5$	1.003	**0.920** (1)**	0.926*** (2)	1.016	1.114	0.790*** (3)	**0.930 (1)**	0.991	**0.762*** (1)**	0.928*	0.998	**0.771*** (1)**
MIN - $\lambda = 1$	0.995	0.930** (3)	**0.925*** (1)**	0.987	1.122	**0.780*** (1)**	0.956	0.989	0.778*** (2)	**0.909*** (1)**	1.000	0.791*** (2)

Notes: Bold numbers indicate lowest CRPS ratios for each horizon and target variable (and therefore best performing models over the full hold-out sample in terms of density forecasts), while the numbers in parenthesis refer to the ranking of the three best specifications. Gray shaded rows denote non-sparsified models. Asterisks indicate statistical significance for each model relative to the benchmark at the 1 (***), 5 (**) and 10 (*) percent significance levels.

References

Clark TE (2011) Real-Time Density Forecasts From Bayesian Vector Autoregressions With Stochastic Volatility. *Journal of Business & Economic Statistics* 29(3), 327–341

McCracken MW and Ng S (2016) FRED-MD: A Monthly Database for Macroeconomic Research. *Journal of Business & Economic Statistics* 34(4), 574–589

Ray P and Bhattacharya A (2018) Signal Adaptive Variable Selector for the Horseshoe Prior. *arXiv:1810.09004*
